# Supplementary figures and images for: The Alternative Splicing Landscape of Brassica napus Infected with Leptosphaeria maculans
Source: Genes (Basel). 2019 Apr 11;10(4):296. doi: 10.3390/genes10040296 (PMC6523698; doi:10.3390/genes10040296)

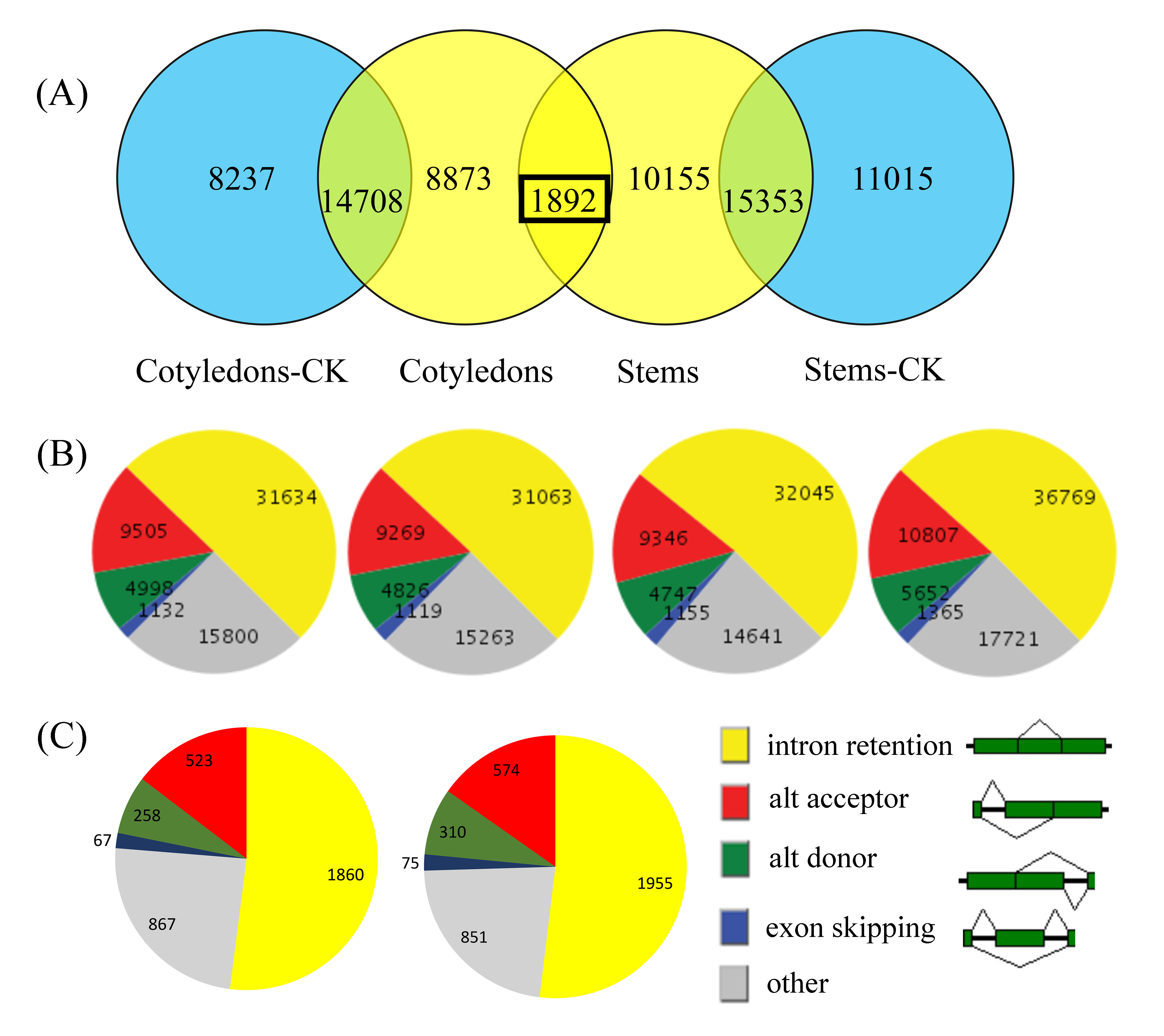

Supplement: Supplementary file 1 [file genes-10-00296-s001.zip › Figure 1.tif]

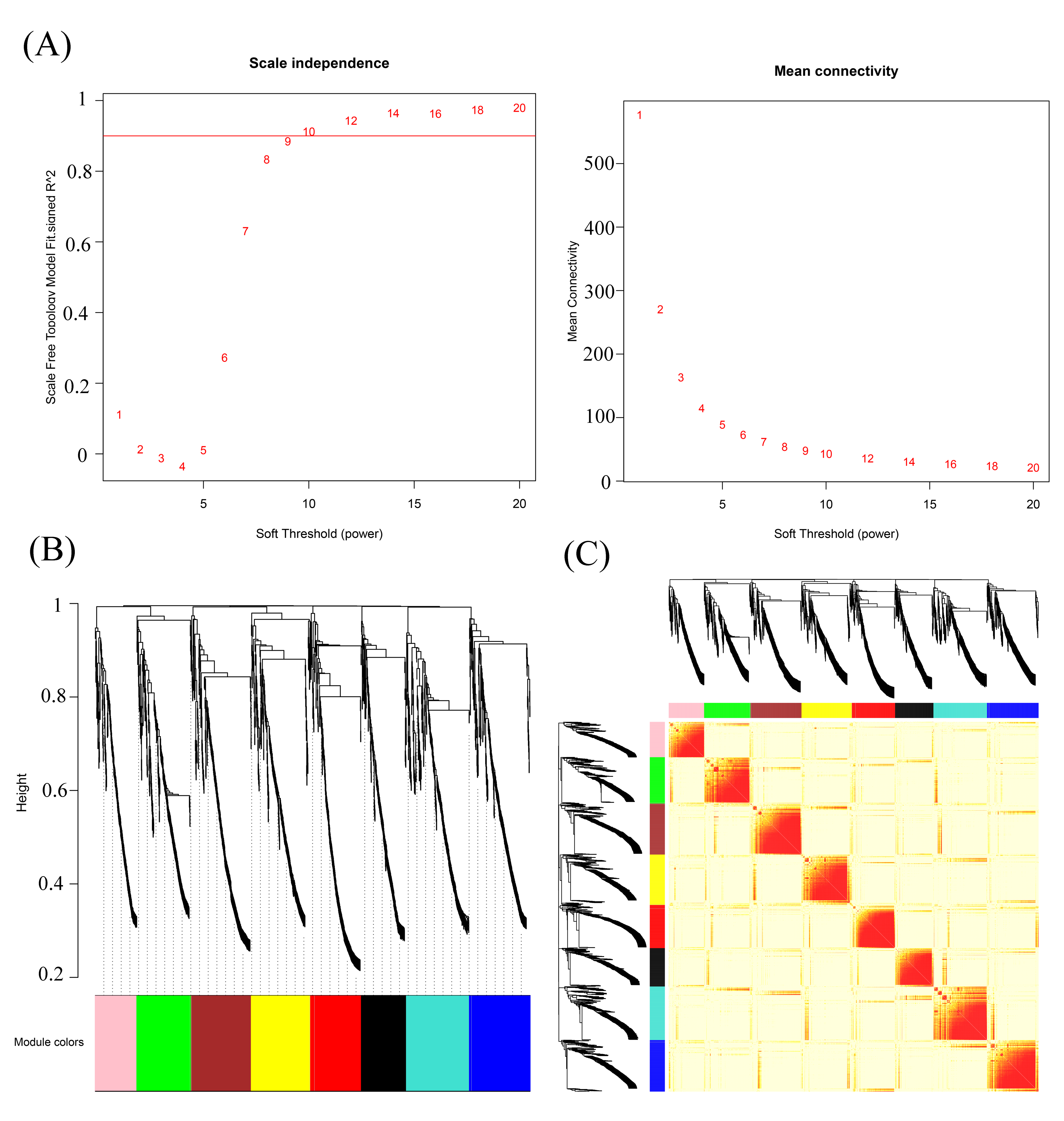

Supplement: Supplementary file 1 [file genes-10-00296-s001.zip › Figure 2.tif]

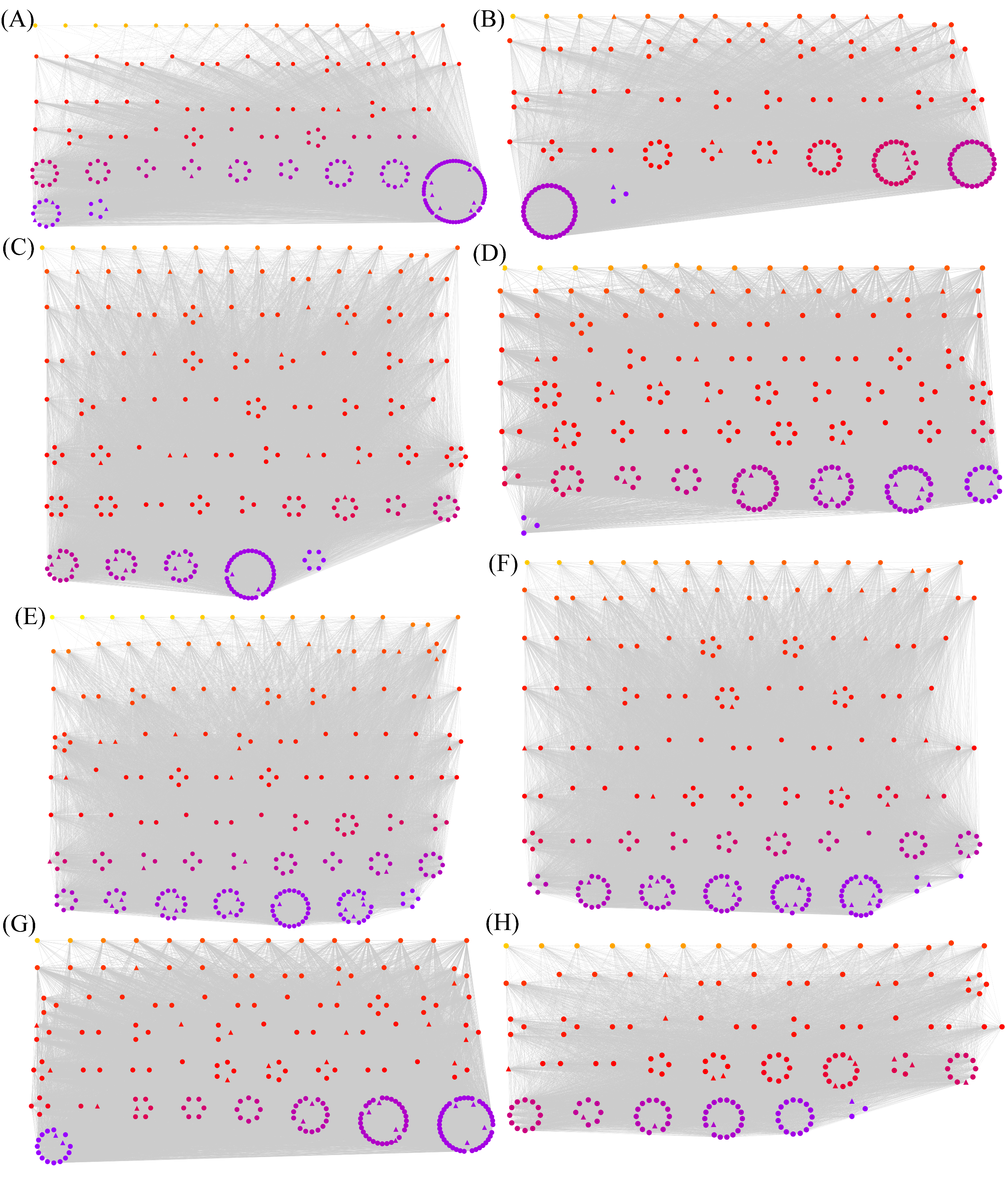

Supplement: Supplementary file 1 [file genes-10-00296-s001.zip › Figure 3.tif]

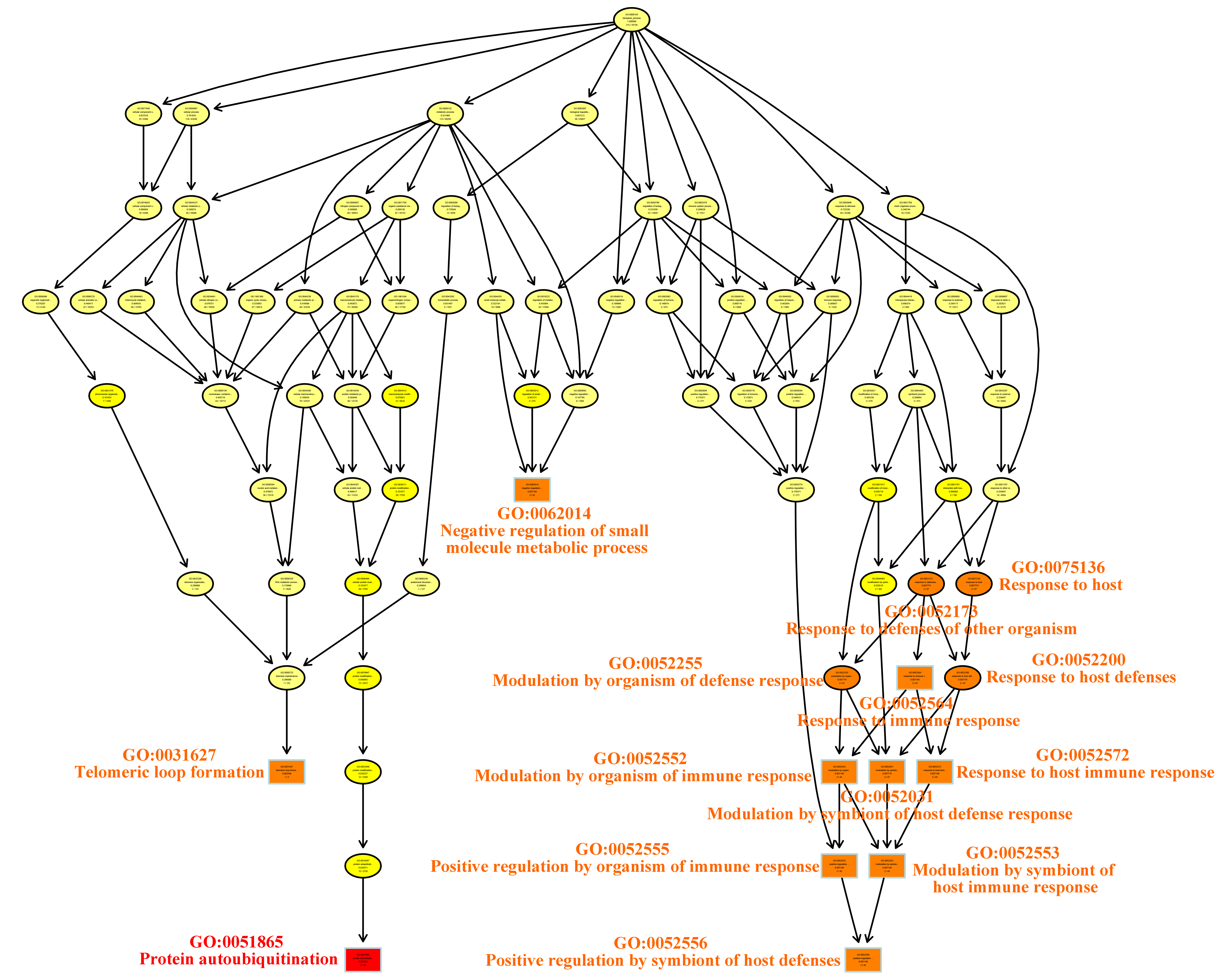

Supplement: Supplementary file 1 [file genes-10-00296-s001.zip › Figure 4.tif]

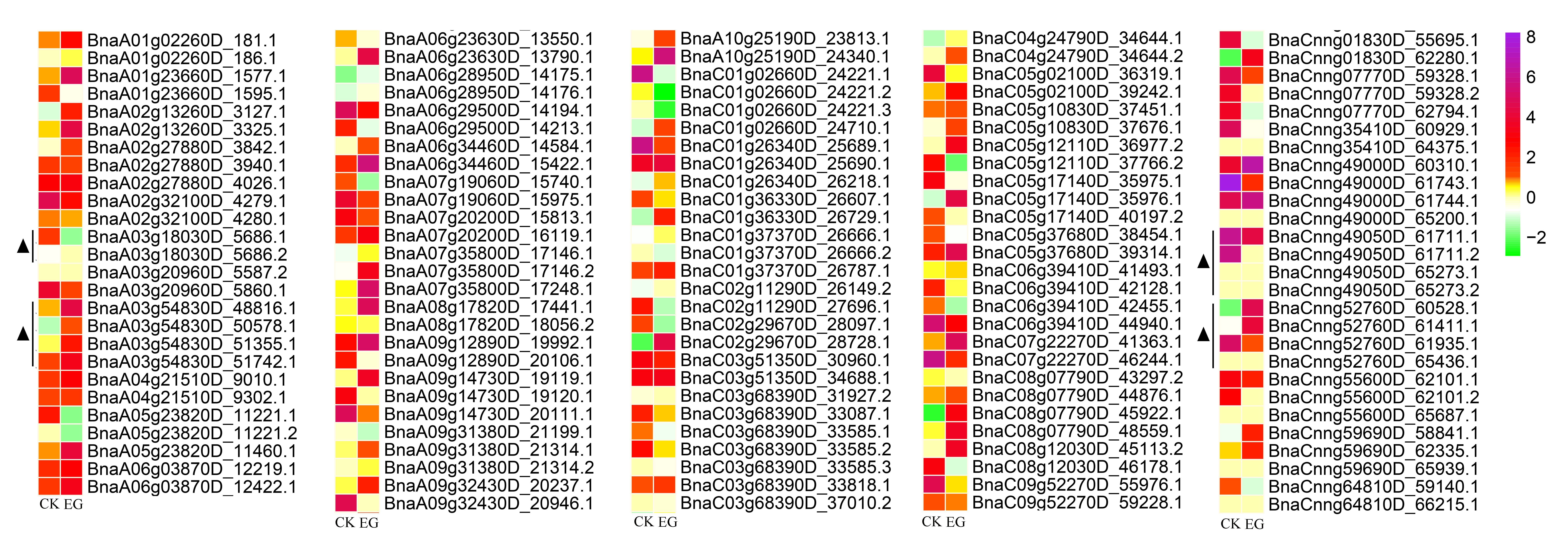

Supplement: Supplementary file 1 [file genes-10-00296-s001.zip › Figure 5A.tif]

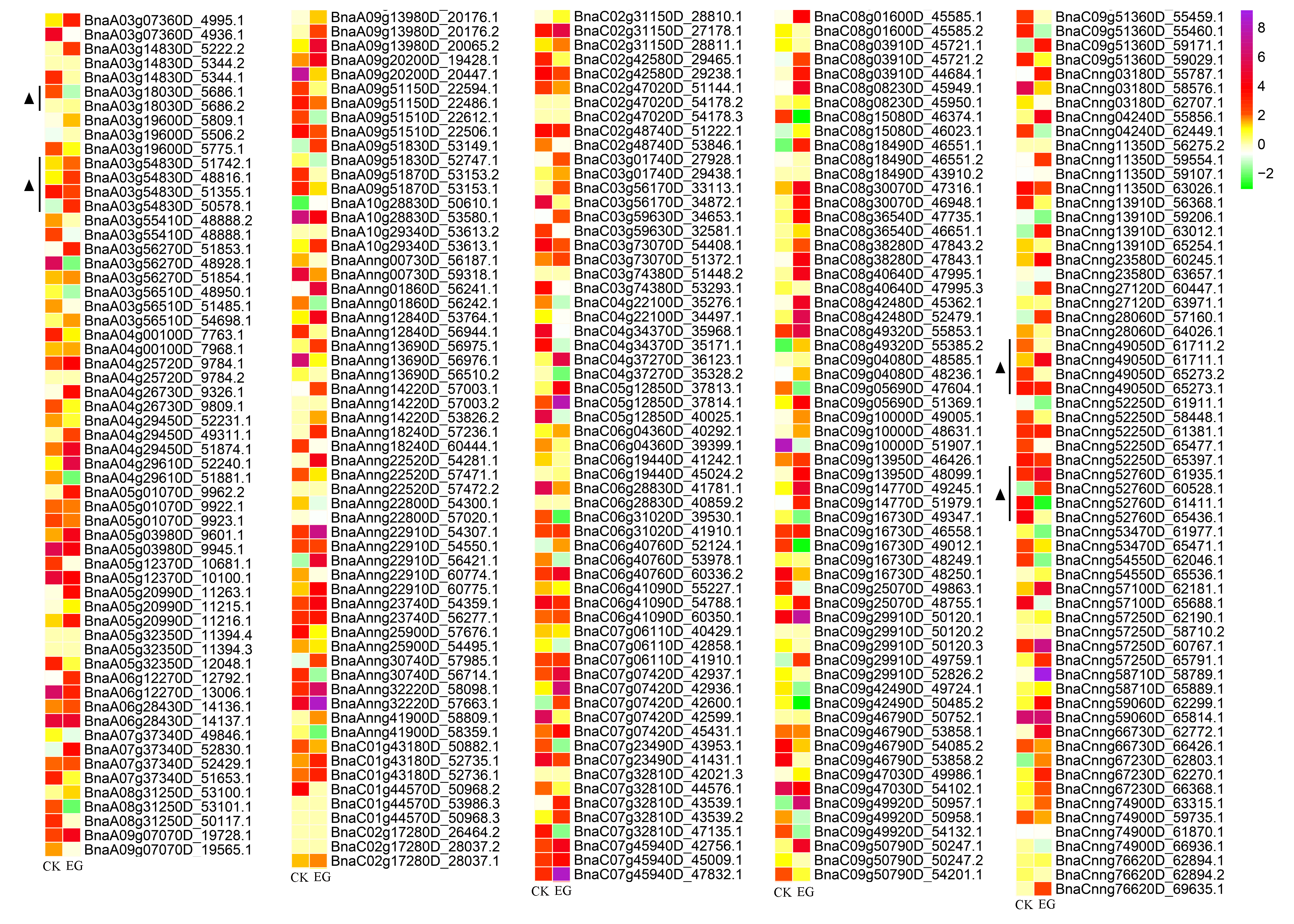

Supplement: Supplementary file 1 [file genes-10-00296-s001.zip › Figure 5B.tif]
