# Supplementary material for: The Alternative Splicing Landscape of Brassica napus Infected with Leptosphaeria maculans
Source: Genes (Basel). 2019 Apr 11;10(4):296. doi: 10.3390/genes10040296 (PMC6523698; doi:10.3390/genes10040296)
Supplement: Supplementary file 1 [file genes-10-00296-s001.zip › Fig. S1.zip!/Fig. S1-1.pdf]

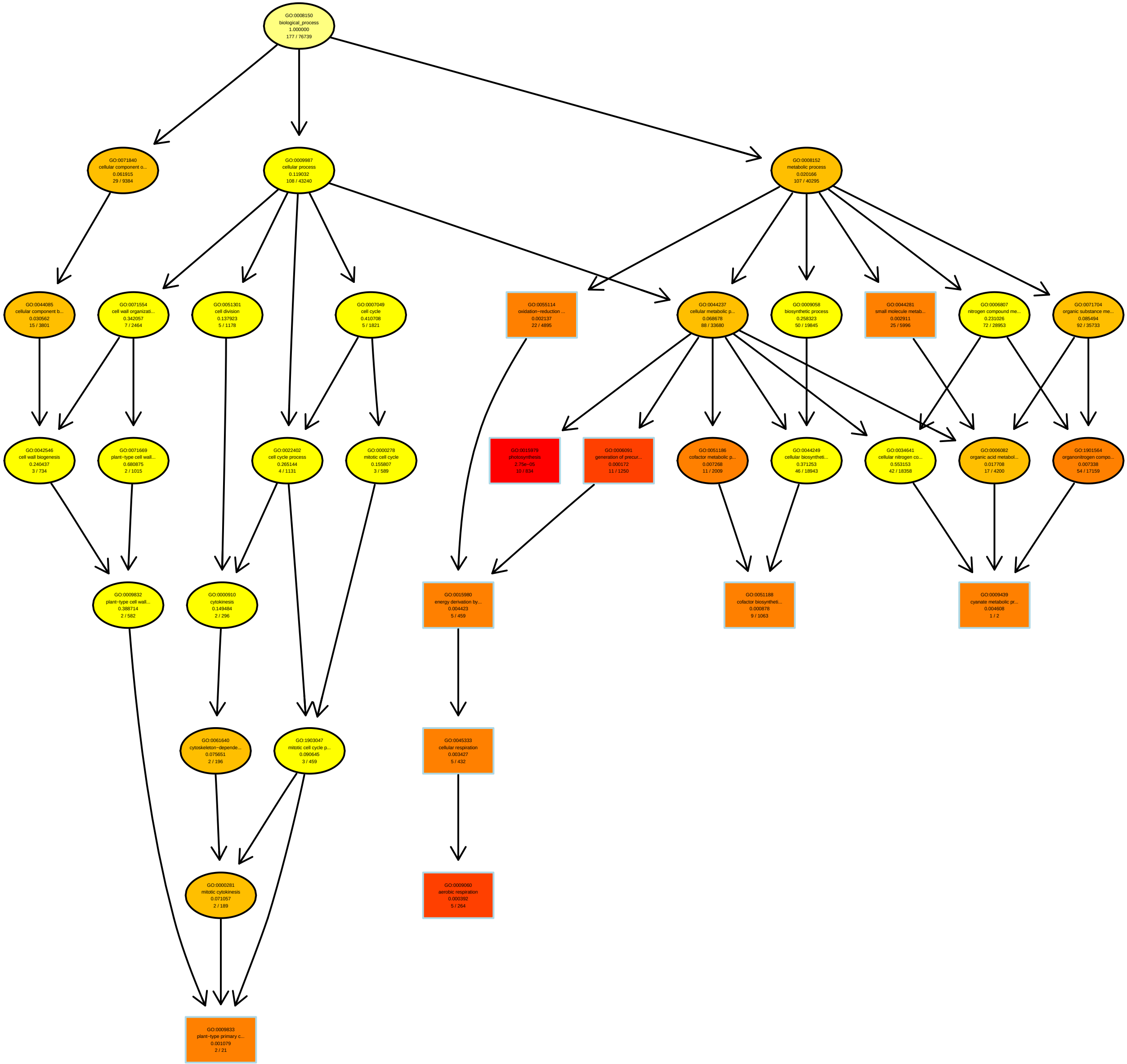
GO:0008150
biological_process
1.000000
177 / 76739
GO:0071840
cellular component o...
0.061915
29 / 9384
GO:0009987
cellular process
0.119032
108 / 43240
GO:0008152
metabolic process
0.020166
107 / 40295
GO:0044085
cellular component b...
0.030562
15 / 3801
GO:0071554
cell wall organizati...
0.342057
7 / 2464
GO:0051301
cell division
0.137923
5 / 1178
GO:0007049
cell cycle
0.410708
5 / 1821
GO:0055114
oxidation−reduction ...
0.002137
22 / 4895
GO:0044237
cellular metabolic p...
0.068678
88 / 33680
GO:0009058
biosynthetic process
0.258323
50 / 19845
GO:0044281
small molecule metab...
0.002911
25 / 5996
GO:0006807
nitrogen compound me...
0.231026
72 / 28953
GO:0071704
organic substance me...
0.085494
92 / 35733
GO:0042546
cell wall biogenesis
0.240437
3 / 734
GO:0071669
plant−type cell wall...
0.680875
2 / 1015
GO:0022402
cell cycle process
0.265144
4 / 1131
GO:0000278
mitotic cell cycle
0.155807
3 / 589
GO:0015979
photosynthesis
2.75e−05
10 / 834
GO:0006091
generation of precur...
0.000172
11 / 1250
GO:0051186
cofactor metabolic p...
0.007268
11 / 2009
GO:0044249
cellular biosyntheti...
0.371253
46 / 18943
GO:0034641
cellular nitrogen co...
0.553153
42 / 18358
GO:0006082
organic acid metabol...
0.017708
17 / 4200
GO:1901564
organonitrogen compo...
0.007338
54 / 17159
GO:0009832
plant−type cell wall...
0.388714
2 / 582
GO:0000910
cytokinesis
0.149484
2 / 296
GO:0015980
energy derivation by...
0.004423
5 / 459
GO:0051188
cofactor biosyntheti...
0.000878
9 / 1063
GO:0009439
cyanate metabolic pr...
0.004608
1 / 2
GO:0061640
cytoskeleton−depende...
0.075651
2 / 196
GO:1903047
mitotic cell cycle p...
0.090645
3 / 459
GO:0045333
cellular respiration
0.003427
5 / 432
GO:0000281
mitotic cytokinesis
0.071057
2 / 189
GO:0009060
aerobic respiration
0.000392
5 / 264
GO:0009833
plant−type primary c...
0.001079
2 / 21

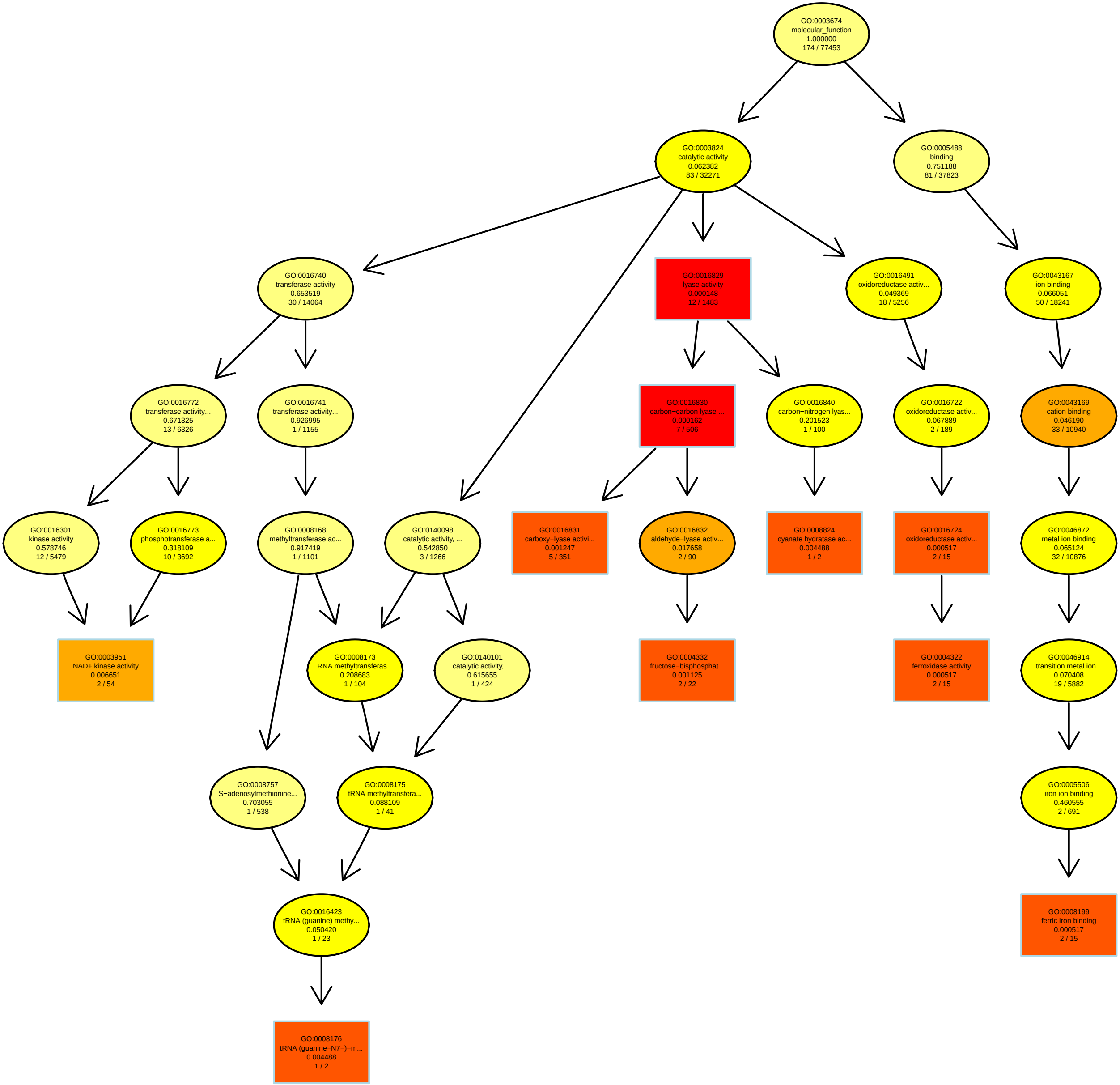
GO:0003674 molecular_function 1.000000 174 / 77453
GO:0003824 catalytic activity 0.062382 83 / 32271
GO:0005488 binding 0.751188 81 / 37823
GO:0016740 transferase activity 0.653519 30 / 14064
GO:0016829 lyase activity 0.000148 12 / 1483
GO:0016491 oxidoreductase activ... 0.049369 18 / 5256
GO:0043167 ion binding 0.066051 50 / 18241
GO:0016772 transferase activity... 0.671325 13 / 6326
GO:0016741 transferase activity... 0.926995 1 / 1155
GO:0016830 carbon−carbon lyase ... 0.000162 7 / 506
GO:0016840 carbon−nitrogen lyas... 0.201523 1 / 100
GO:0016722 oxidoreductase activ... 0.067889 2 / 189
GO:0043169 cation binding 0.046190 33 / 10940
GO:0016301 kinase activity 0.578746 12 / 5479
GO:0016773 phosphotransferase a... 0.318109 10 / 3692
GO:0008168 methyltransferase ac... 0.917419 1 / 1101
GO:0140098 catalytic activity, ... 0.542850 3 / 1266
GO:0016831 carboxy−lyase activi... 0.001247 5 / 351
GO:0016832 aldehyde−lyase activ... 0.017658 2 / 90
GO:0008824 cyanate hydratase ac... 0.004488 1 / 2
GO:0016724 oxidoreductase activ... 0.000517 2 / 15
GO:0046872 metal ion binding 0.065124 32 / 10876
GO:0003951 NAD+ kinase activity 0.006651 2 / 54
GO:0008173 RNA methyltransferas... 0.208683 1 / 104
GO:0140101 catalytic activity, ... 0.615655 1 / 424
GO:0004332 fructose−bisphosphat... 0.001125 2 / 22
GO:0004322 ferroxidase activity 0.000517 2 / 15
GO:0046914 transition metal ion... 0.070408 19 / 5882
GO:0008757 S−adenosylmethionine... 0.703055 1 / 538
GO:0008175 tRNA methyltransfera... 0.088109 1 / 41
GO:0005506 iron ion binding 0.460555 2 / 691
GO:0016423 tRNA (guanine) methy... 0.050420 1 / 23
GO:0008199 ferric iron binding 0.000517 2 / 15
GO:0008176 tRNA (guanine−N7−)−m... 0.004488 1 / 2

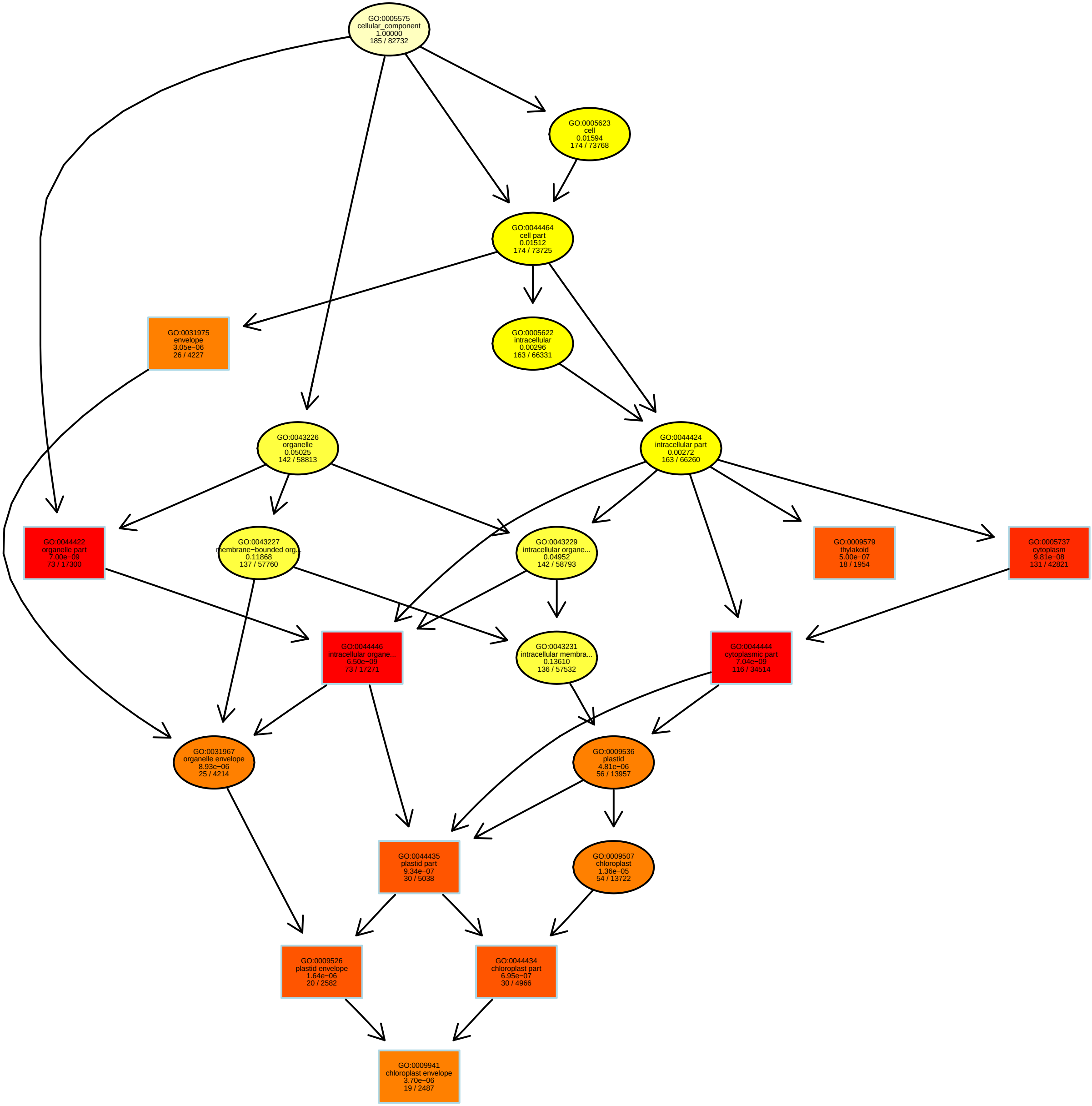
GO:0005575
cellular_component
1.00000
185 / 82732
GO:0005623
cell
0.01594
174 / 73768
GO:0044464
cell part
0.01512
174 / 73725
GO:0031975
envelope
3.05e−06
26 / 4227
GO:0005622
intracellular
0.00296
163 / 66331
GO:0043226
organelle
0.05025
142 / 58813
GO:0044424
intracellular part
0.00272
163 / 66260
GO:0044422
organelle part
7.00e−09
73 / 17300
GO:0043227
membrane−bounded org...
0.11868
137 / 57760
GO:0043229
intracellular organe...
0.04952
142 / 58793
GO:0009579
thylakoid
5.00e−07
18 / 1954
GO:0005737
cytoplasm
9.81e−08
131 / 42821
GO:0044446
intracellular organe...
6.50e−09
73 / 17271
GO:0043231
intracellular membra...
0.13610
136 / 57532
GO:0044444
cytoplasmic part
7.04e−09
116 / 34514
GO:0031967
organelle envelope
8.93e−06
25 / 4214
GO:0009536
plastid
4.81e−06
56 / 13957
GO:0044435
plastid part
9.34e−07
30 / 5038
GO:0009507
chloroplast
1.36e−05
54 / 13722
GO:0009526
plastid envelope
1.64e−06
20 / 2582
GO:0044434
chloroplast part
6.95e−07
30 / 4966
GO:0009941
chloroplast envelope
3.70e−06
19 / 2487
