# Supplementary material for: The Alternative Splicing Landscape of Brassica napus Infected with Leptosphaeria maculans
Source: Genes (Basel). 2019 Apr 11;10(4):296. doi: 10.3390/genes10040296 (PMC6523698; doi:10.3390/genes10040296)
Supplement: Supplementary file 1 [file genes-10-00296-s001.zip › Fig. S1.zip!/Fig. S1-2.pdf]

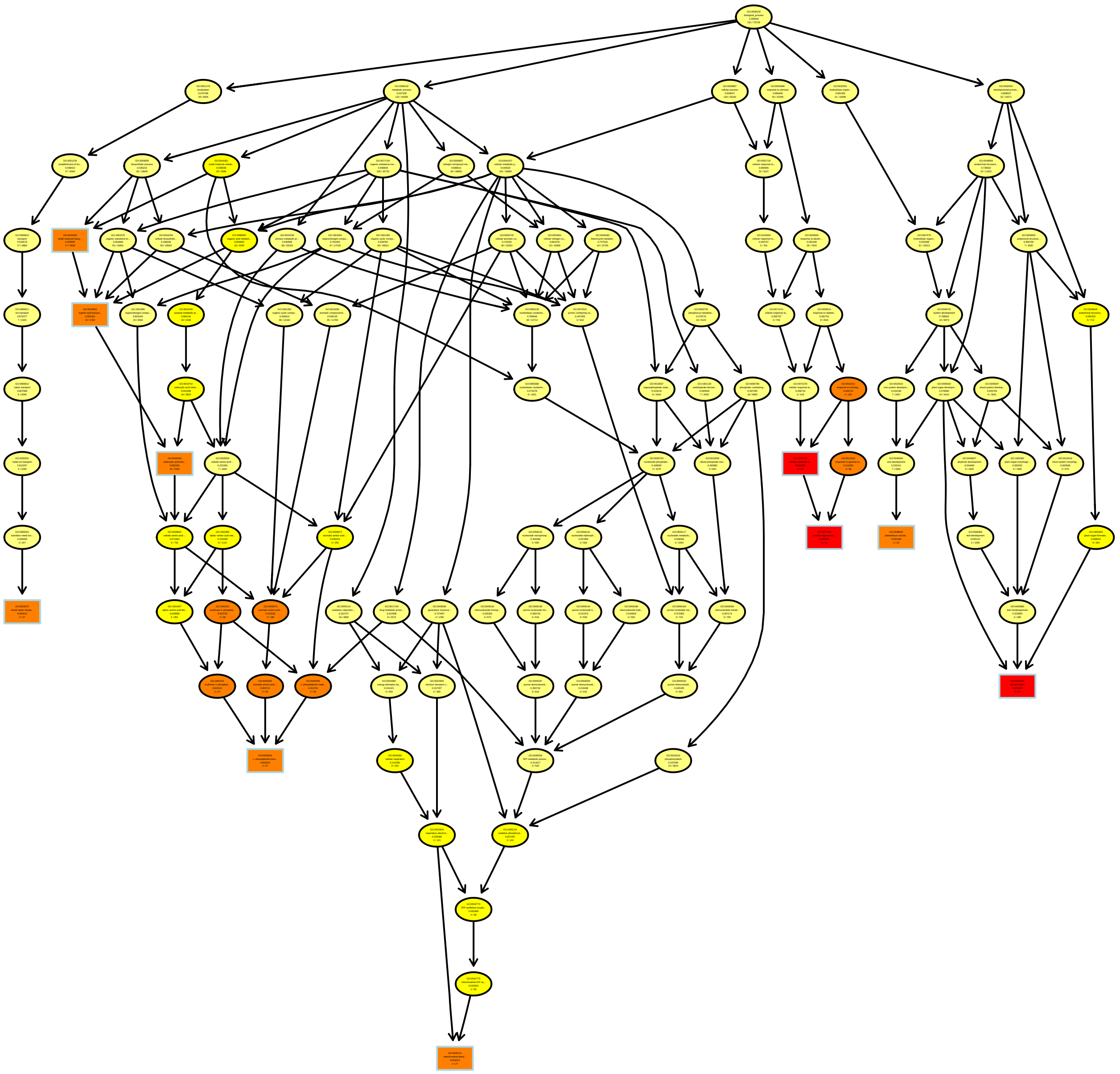

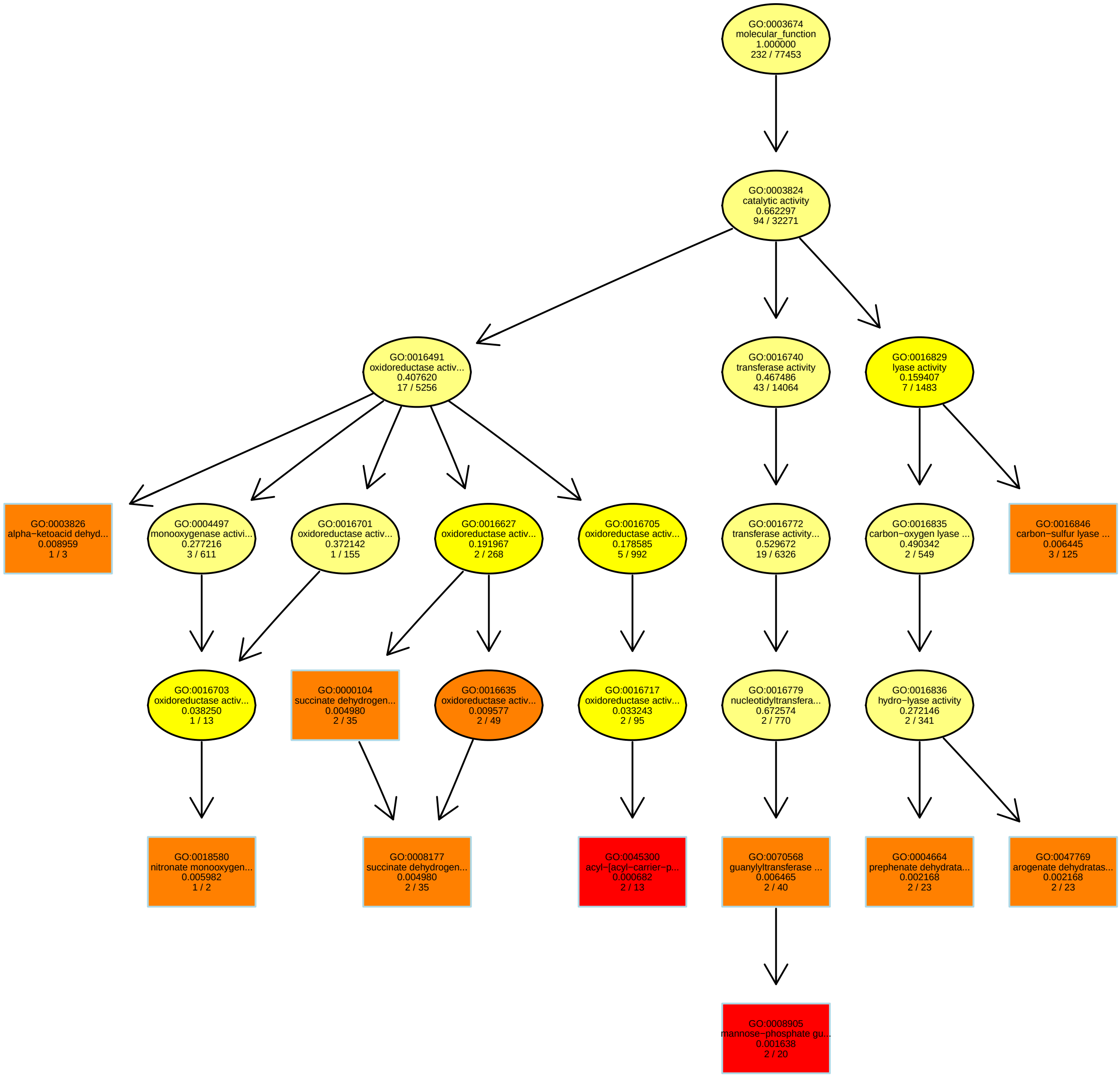

GO:0003674
molecular_function
1.000000
232 / 77453
GO:0003824
catalytic activity
0.662297
94 / 32271
GO:0016491
oxidoreductase activ...
0.407620
17 / 5256
GO:0016740
transferase activity
0.467486
43 / 14064
GO:0016829
lyase activity
0.159407
7 / 1483
GO:0003826
alpha−ketoacid dehyd...
0.008959
1 / 3
GO:0004497
monooxygenase activi...
0.277216
3 / 611
GO:0016701
oxidoreductase activ...
0.372142
1 / 155
GO:0016627
oxidoreductase activ...
0.191967
2 / 268
GO:0016705
oxidoreductase activ...
0.178585
5 / 992
GO:0016772
transferase activity...
0.529672
19 / 6326
GO:0016835
carbon−oxygen lyase ...
0.490342
2 / 549
GO:0016846
carbon−sulfur lyase ...
0.006445
3 / 125
GO:0016703
oxidoreductase activ...
0.038250
1 / 13
GO:0000104
succinate dehydrogen...
0.004980
2 / 35
GO:0016635
oxidoreductase activ...
0.009577
2 / 49
GO:0016717
oxidoreductase activ...
0.033243
2 / 95
GO:0016779
nucleotidyltransfera...
0.672574
2 / 770
GO:0016836
hydro−lyase activity
0.272146
2 / 341
GO:0018580
nitronate monooxygen...
0.005982
1 / 2
GO:0008177
succinate dehydrogen...
0.004980
2 / 35
GO:0045300
acyl−[acyl−carrier−p...
0.000682
2 / 13
GO:0070568
guanylyltransferase ...
0.006465
2 / 40
GO:0004664
prephenate dehydrata...
0.002168
2 / 23
GO:0047769
arogenate dehydratas...
0.002168
2 / 23
GO:0008905
mannose−phosphate gu...
0.001638
2 / 20

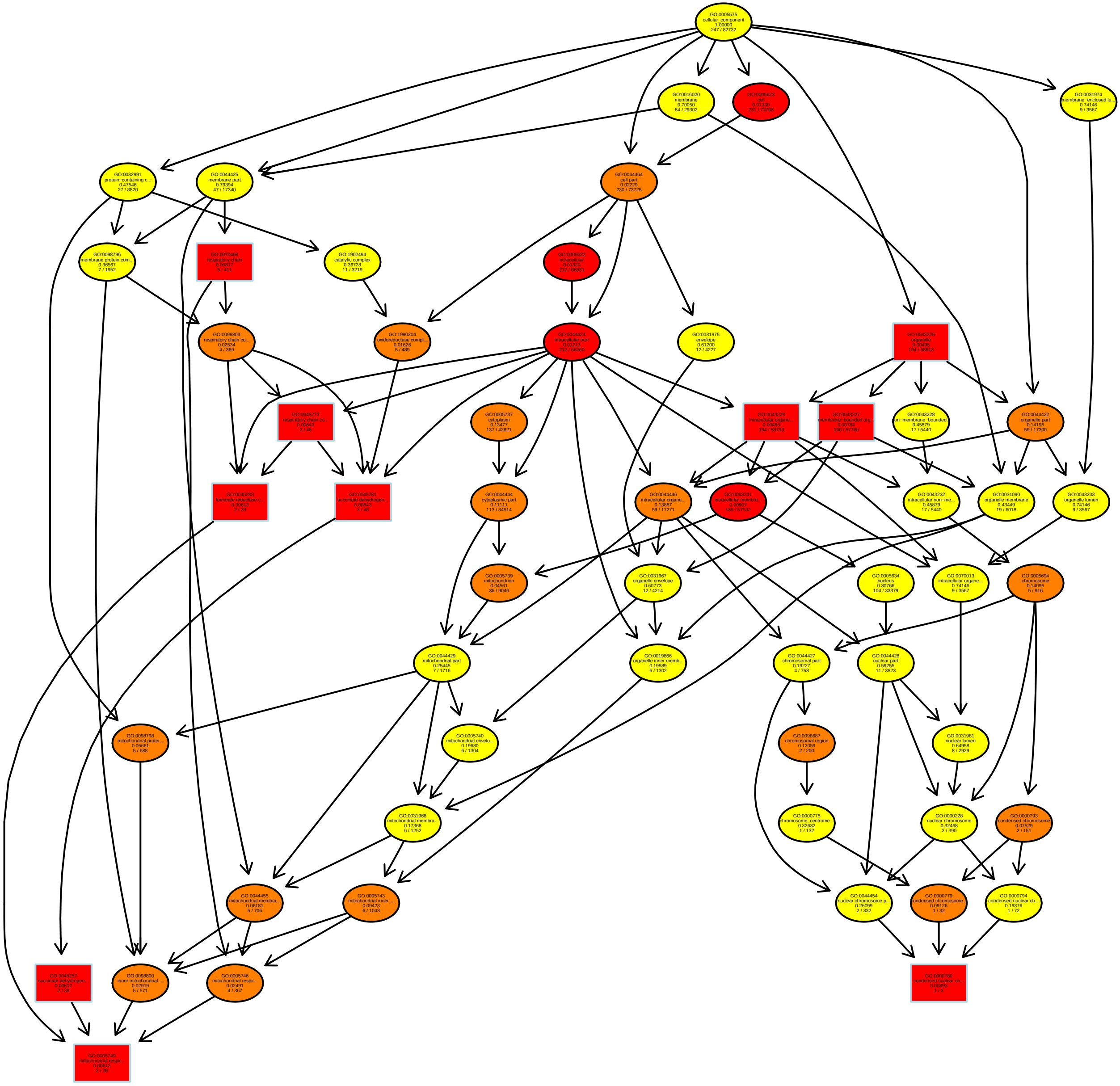
GO:0005575 cellular_component 1.00000 247 / 82732
GO:0016020 membrane 0.70050 84 / 29302
GO:0005623 cell 0.01330 231 / 73768
GO:0031974 membrane−enclosed lu... 0.74146 9 / 3567
GO:0032991 protein−containing c... 0.47546 27 / 8820
GO:0044425 membrane part 0.79394 47 / 17340
GO:0044464 cell part 0.02229 230 / 73725
GO:0098796 membrane protein com... 0.36567 7 / 1952
GO:0070469 respiratory chain 0.00817 5 / 411
GO:1902494 catalytic complex 0.36728 11 / 3219
GO:0005622 intracellular 0.01320 212 / 66331
GO:0098803 respiratory chain co... 0.02534 4 / 369
GO:1990204 oxidoreductase compl... 0.01626 5 / 489
GO:0044424 intracellular part 0.01213 212 / 66260
GO:0031975 envelope 0.61200 12 / 4227
GO:0043226 organelle 0.00495 194 / 58813
GO:0045273 respiratory chain co... 0.00843 2 / 46
GO:0005737 cytoplasm 0.13477 137 / 42821
GO:0043229 intracellular organe... 0.00483 194 / 58793
GO:0043227 membrane−bounded org... 0.00784 190 / 57760
GO:0043228 on−membrane−bounded... 0.45879 17 / 5440
GO:0044422 organelle part 0.14195 59 / 17300
GO:0045283 fumarate reductase c... 0.00612 2 / 39
GO:0045281 succinate dehydrogen... 0.00843 2 / 46
GO:0044444 cytoplasmic part 0.11111 113 / 34514
GO:0044446 intracellular organe... 0.13887 59 / 17271
GO:0043231 intracellular membra... 0.00907 189 / 57532
GO:0043232 intracellular non−me... 0.45879 17 / 5440
GO:0031090 organelle membrane 0.43449 19 / 6018
GO:0043233 organelle lumen 0.74146 9 / 3567
GO:0005739 mitochondrion 0.04561 36 / 9046
GO:0031967 organelle envelope 0.60773 12 / 4214
GO:0005634 nucleus 0.30766 104 / 33379
GO:0070013 intracellular organe... 0.74146 9 / 3567
GO:0005694 chromosome 0.14095 5 / 916
GO:0044429 mitochondrial part 0.25445 7 / 1716
GO:0019866 organelle inner memb... 0.19589 6 / 1302
GO:0044427 chromosomal part 0.19227 4 / 758
GO:0044428 nuclear part 0.59255 11 / 3823
GO:0098798 mitochondrial protei... 0.05661 5 / 688
GO:0005740 mitochondrial envelo... 0.19680 6 / 1304
GO:0098687 chromosomal region 0.12059 2 / 200
GO:0031981 nuclear lumen 0.64958 8 / 2929
GO:0031966 mitochondrial membra... 0.17368 6 / 1252
GO:0000775 chromosome, centrome... 0.32632 1 / 132
GO:0000228 nuclear chromosome 0.32468 2 / 390
GO:0000793 condensed chromosome 0.07529 2 / 151
GO:0044455 mitochondrial membra... 0.06181 5 / 706
GO:0005743 mitochondrial inner ... 0.09423 6 / 1043
GO:0044454 nuclear chromosome p... 0.26099 2 / 332
GO:0000779 ondensed chromosome... 0.09126 1 / 32
GO:0000794 condensed nuclear ch... 0.19376 1 / 72
GO:0045257 succinate dehydrogen... 0.00612 2 / 39
GO:0098800 inner mitochondrial ... 0.02919 5 / 571
GO:0005746 mitochondrial respir... 0.02491 4 / 367
GO:0000780 condensed nuclear ch... 0.00893 1 / 3
GO:0005749 mitochondrial respir... 0.00612 2 / 39
