# Supplementary material for: The Alternative Splicing Landscape of Brassica napus Infected with Leptosphaeria maculans
Source: Genes (Basel). 2019 Apr 11;10(4):296. doi: 10.3390/genes10040296 (PMC6523698; doi:10.3390/genes10040296)
Supplement: Supplementary file 1 [file genes-10-00296-s001.zip › Fig. S1.zip!/Fig. S1-3.pdf]

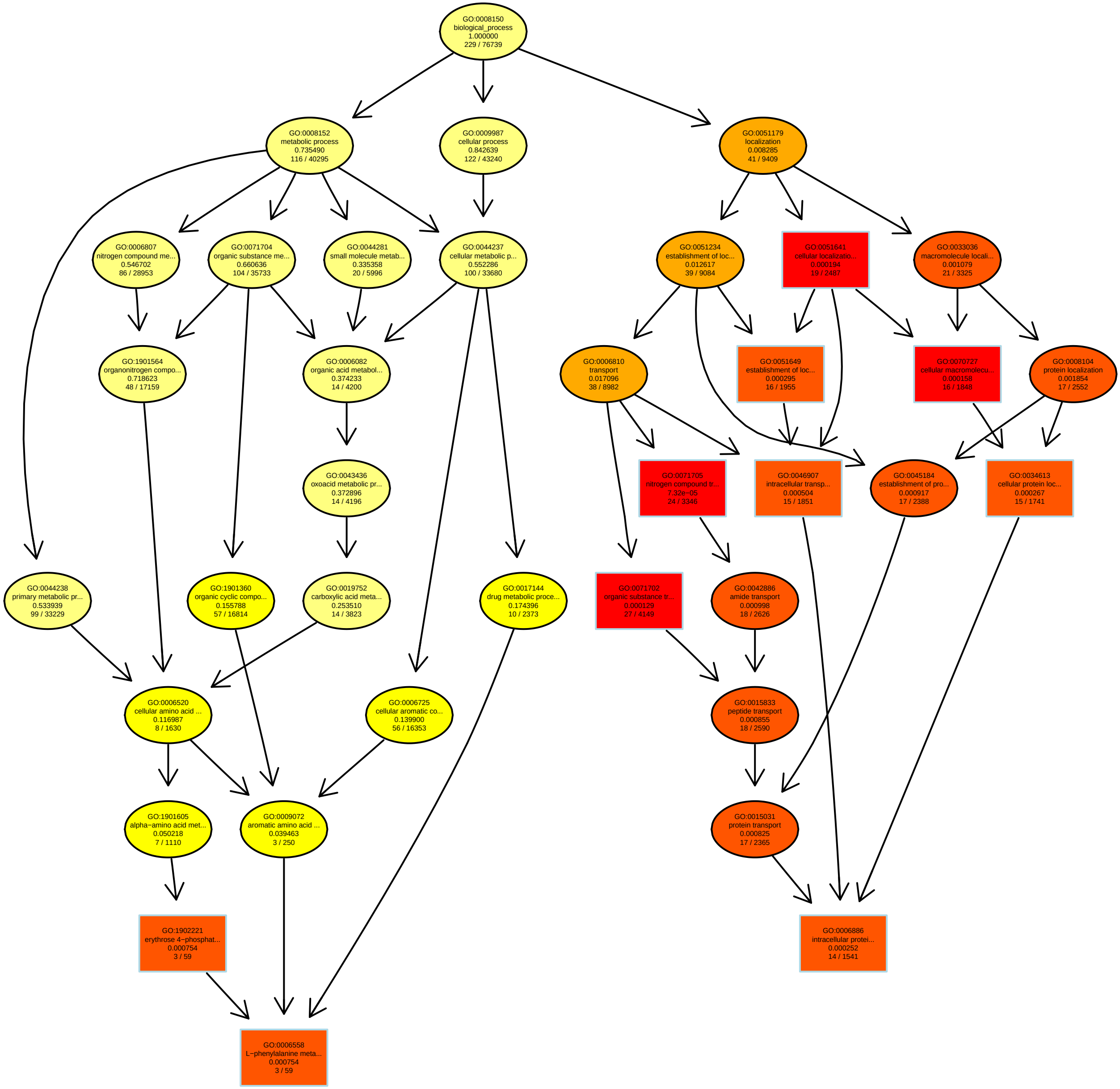
GO:0008150
biological_process
1.000000
229 / 76739
GO:0008152
metabolic process
0.735490
116 / 40295
GO:0009987
cellular process
0.842639
122 / 43240
GO:0051179
localization
0.008285
41 / 9409
GO:0006807
nitrogen compound me...
0.546702
86 / 28953
GO:0071704
organic substance me...
0.660636
104 / 35733
GO:0044281
small molecule metab...
0.335358
20 / 5996
GO:0044237
cellular metabolic p...
0.552286
100 / 33680
GO:0051234
establishment of loc...
0.012617
39 / 9084
GO:0051641
cellular localizatio...
0.000194
19 / 2487
GO:0033036
macromolecule locali...
0.001079
21 / 3325
GO:1901564
organonitrogen compo...
0.718623
48 / 17159
GO:0006082
organic acid metabol...
0.374233
14 / 4200
GO:0006810
transport
0.017096
38 / 8982
GO:0051649
establishment of loc...
0.000295
16 / 1955
GO:0070727
cellular macromolecu...
0.000158
16 / 1848
GO:0008104
protein localization
0.001854
17 / 2552
GO:0043436
oxoacid metabolic pr...
0.372896
14 / 4196
GO:0071705
nitrogen compound tr...
7.32e−05
24 / 3346
GO:0046907
intracellular transp...
0.000504
15 / 1851
GO:0045184
establishment of pro...
0.000917
17 / 2388
GO:0034613
cellular protein loc...
0.000267
15 / 1741
GO:0044238
primary metabolic pr...
0.533939
99 / 33229
GO:1901360
organic cyclic compo...
0.155788
57 / 16814
GO:0019752
carboxylic acid meta...
0.253510
14 / 3823
GO:0017144
drug metabolic proce...
0.174396
10 / 2373
GO:0071702
organic substance tr...
0.000129
27 / 4149
GO:0042886
amide transport
0.000998
18 / 2626
GO:0006520
cellular amino acid ...
0.116987
8 / 1630
GO:0006725
cellular aromatic co...
0.139900
56 / 16353
GO:0015833
peptide transport
0.000855
18 / 2590
GO:1901605
alpha−amino acid met...
0.050218
7 / 1110
GO:0009072
aromatic amino acid ...
0.039463
3 / 250
GO:0015031
protein transport
0.000825
17 / 2365
GO:1902221
erythrose 4−phosphat...
0.000754
3 / 59
GO:0006886
intracellular protei...
0.000252
14 / 1541
GO:0006558
L−phenylalanine meta...
0.000754
3 / 59

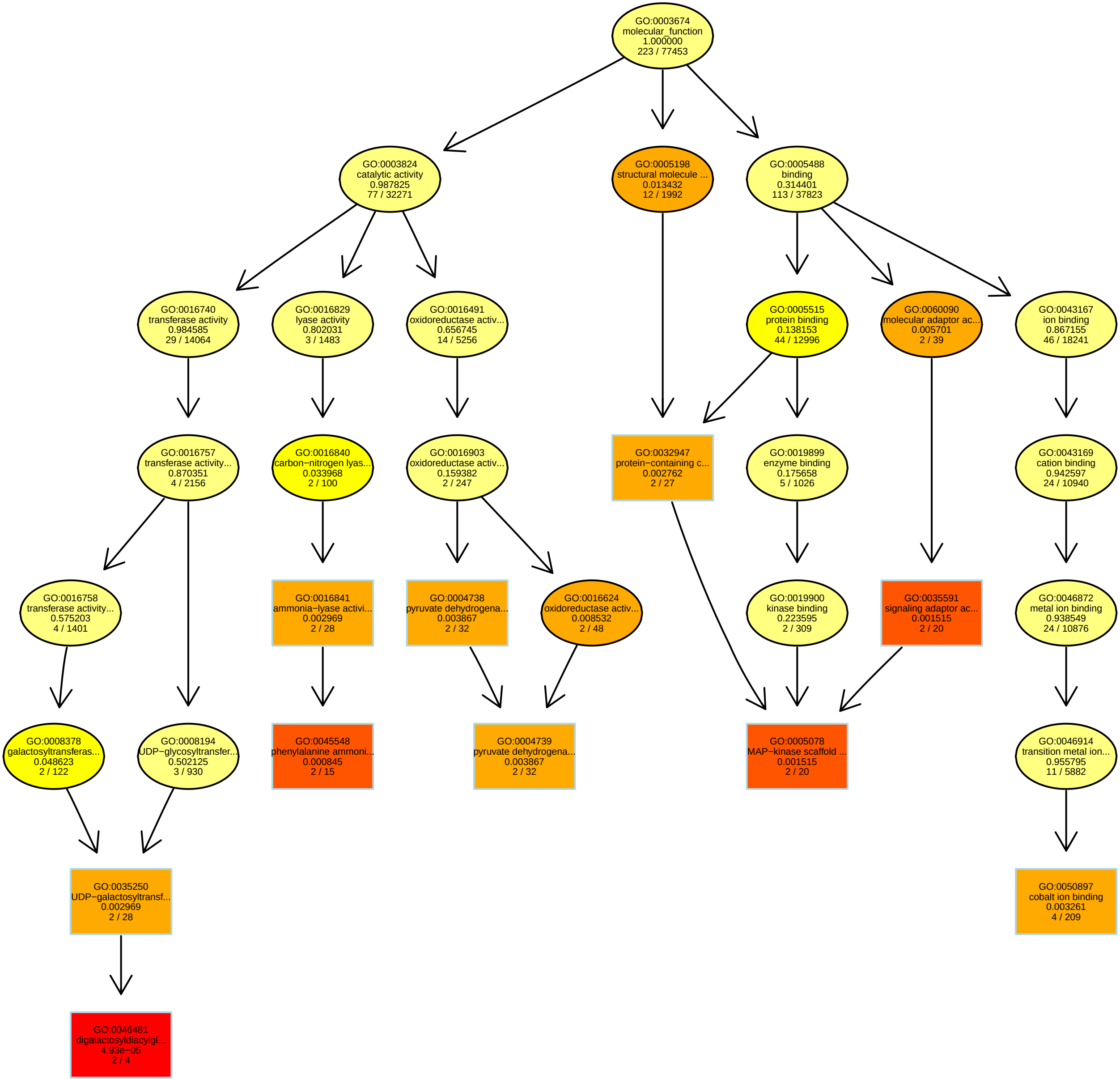
GO:0003674
molecular_function
1.000000
223 / 77453
GO:0003824
catalytic activity
0.987825
77 / 32271
GO:0005198
structural molecule ...
0.013432
12 / 1992
GO:0005488
binding
0.314401
113 / 37823
GO:0016740
transferase activity
0.984585
29 / 14064
GO:0016829
lyase activity
0.802031
3 / 1483
GO:0016491
oxidoreductase activ...
0.656745
14 / 5256
GO:0005515
protein binding
0.138153
44 / 12996
GO:0060090
molecular adaptor ac...
0.005701
2 / 39
GO:0043167
ion binding
0.867155
46 / 18241
GO:0016757
transferase activity...
0.870351
4 / 2156
GO:0016840
carbon−nitrogen lyas...
0.033968
2 / 100
GO:0016903
oxidoreductase activ...
0.159382
2 / 247
GO:0032947
protein−containing c...
0.002762
2 / 27
GO:0019899
enzyme binding
0.175658
5 / 1026
GO:0043169
cation binding
0.942597
24 / 10940
GO:0016758
transferase activity...
0.575203
4 / 1401
GO:0016841
ammonia−lyase activi...
0.002969
2 / 28
GO:0004738
pyruvate dehydrogena...
0.003867
2 / 32
GO:0016624
oxidoreductase activ...
0.008532
2 / 48
GO:0019900
kinase binding
0.223595
2 / 309
GO:0035591
signaling adaptor ac...
0.001515
2 / 20
GO:0046872
metal ion binding
0.938549
24 / 10876
GO:0008378
galactosyltransferas...
0.048623
2 / 122
GO:0008194
UDP−glycosyltransfer...
0.502125
3 / 930
GO:0045548
phenylalanine ammoni...
0.000845
2 / 15
GO:0004739
pyruvate dehydrogena...
0.003867
2 / 32
GO:0005078
MAP−kinase scaffold ...
0.001515
2 / 20
GO:0046914
transition metal ion...
0.955795
11 / 5882
GO:0035250
UDP−galactosyltransf...
0.002969
2 / 28
GO:0050897
cobalt ion binding
0.003261
4 / 209
GO:0046481
digalactosyldiacylgl...
4.93e−05
2 / 4

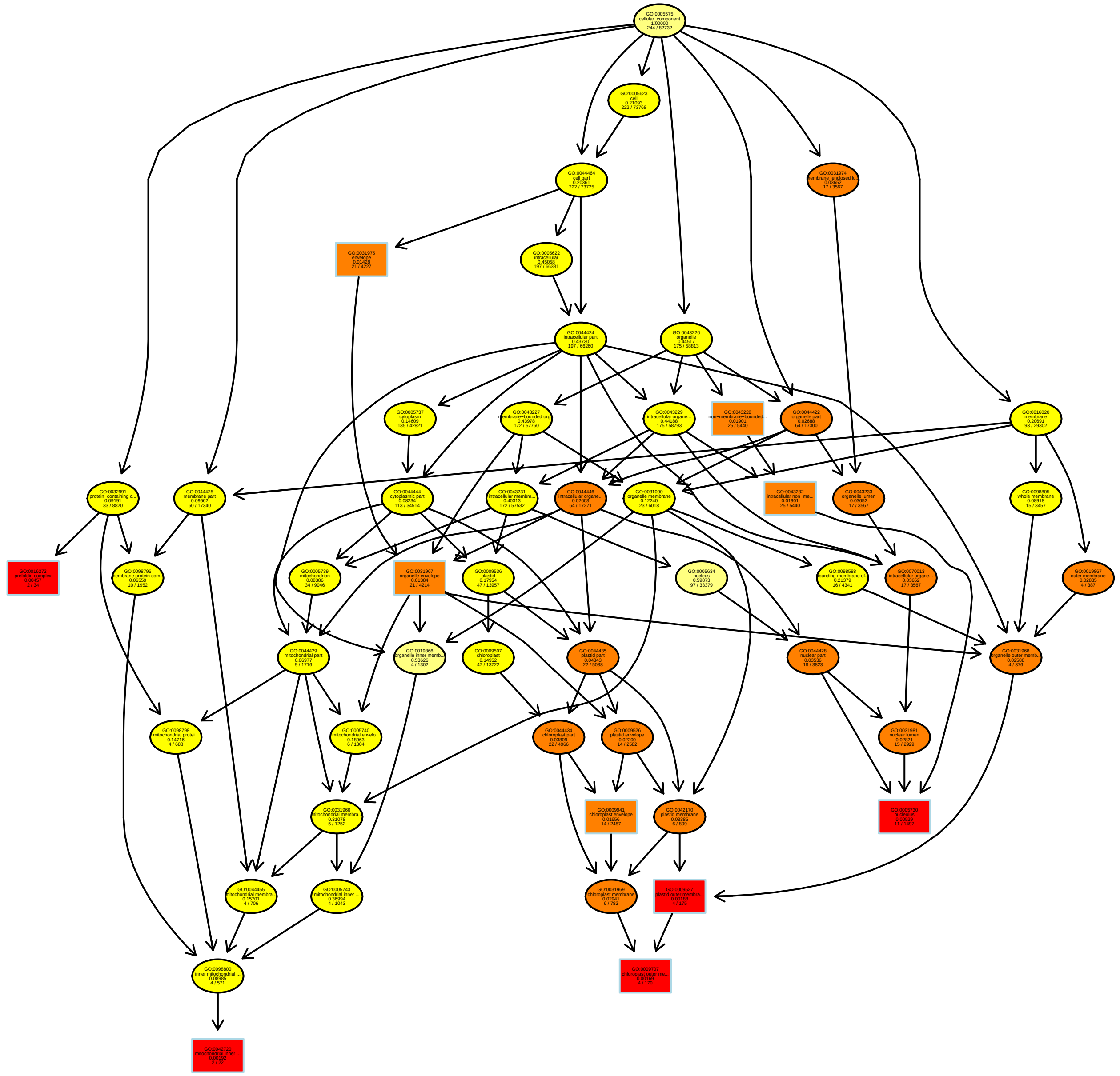

GO:0005575 cellular_component 1.00000 244 / 82732
GO:0005623 cell 0.21093 222 / 73768
GO:0044464 cell part 0.20361 222 / 73725
GO:0031974 membrane−enclosed lu... 0.03652 17 / 3567
GO:0031975 envelope 0.01428 21 / 4227
GO:0005622 intracellular 0.45058 197 / 66331
GO:0044424 intracellular part 0.43730 197 / 66260
GO:0043226 organelle 0.44517 175 / 58813
GO:0005737 cytoplasm 0.14609 135 / 42821
GO:0043227 membrane−bounded org... 0.43978 172 / 57760
GO:0043229 intracellular organe... 0.44188 175 / 58793
GO:0043228 non−membrane−bounded... 0.01901 25 / 5440
GO:0044422 organelle part 0.02688 64 / 17300
GO:0016020 membrane 0.20691 93 / 29302
GO:0032991 protein−containing c... 0.09191 33 / 8820
GO:0044425 membrane part 0.09562 60 / 17340
GO:0044444 cytoplasmic part 0.08234 113 / 34514
GO:0043231 intracellular membra... 0.40313 172 / 57532
GO:0044446 intracellular organe... 0.02603 64 / 17271
GO:0031090 organelle membrane 0.12240 23 / 6018
GO:0043232 intracellular non−me... 0.01901 25 / 5440
GO:0043233 organelle lumen 0.03652 17 / 3567
GO:0098805 whole membrane 0.08918 15 / 3457
GO:0016272 prefoldin complex 0.00457 2 / 34
GO:0098796 membrane protein com... 0.06559 10 / 1952
GO:0005739 mitochondrion 0.08386 34 / 9046
GO:0031967 organelle envelope 0.01384 21 / 4214
GO:0009536 plastid 0.17954 47 / 13957
GO:0005634 nucleus 0.59873 97 / 33379
GO:0098588 bounding membrane of... 0.21379 16 / 4341
GO:0070013 intracellular organe... 0.03652 17 / 3567
GO:0019867 outer membrane 0.02835 4 / 387
GO:0044429 mitochondrial part 0.06977 9 / 1715
GO:0019866 organelle inner memb... 0.53626 4 / 1302
GO:0009507 chloroplast 0.14952 47 / 13722
GO:0044435 plastid part 0.04343 22 / 5038
GO:0044428 nuclear part 0.03536 18 / 3823
GO:0031968 organelle outer memb... 0.02588 4 / 376
GO:0098798 mitochondrial protei... 0.14716 4 / 688
GO:0005740 mitochondrial envelo... 0.18963 6 / 1304
GO:0044434 chloroplast part 0.03809 22 / 4966
GO:0009526 plastid envelope 0.02200 14 / 2582
GO:0031981 nuclear lumen 0.02821 15 / 2929
GO:0031966 mitochondrial membra... 0.31078 5 / 1252
GO:0009941 chloroplast envelope 0.01656 14 / 2487
GO:0042170 plastid membrane 0.03385 6 / 809
GO:0005730 nucleolus 0.00529 11 / 1497
GO:0044455 mitochondrial membra... 0.15701 4 / 706
GO:0005743 mitochondrial inner ... 0.36994 4 / 1043
GO:0031969 chloroplast membrane 0.02941 6 / 782
GO:0009527 plastid outer membra... 0.00188 4 / 175
GO:0098800 inner mitochondrial ... 0.08985 4 / 571
GO:0009707 chloroplast outer me... 0.00160 4 / 170
GO:0042720 mitochondrial inner ... 0.00192 2 / 22
