# Supplementary material for: The Alternative Splicing Landscape of Brassica napus Infected with Leptosphaeria maculans
Source: Genes (Basel). 2019 Apr 11;10(4):296. doi: 10.3390/genes10040296 (PMC6523698; doi:10.3390/genes10040296)
Supplement: Supplementary file 1 [file genes-10-00296-s001.zip › Fig. S1.zip!/Fig. S1-4.pdf]

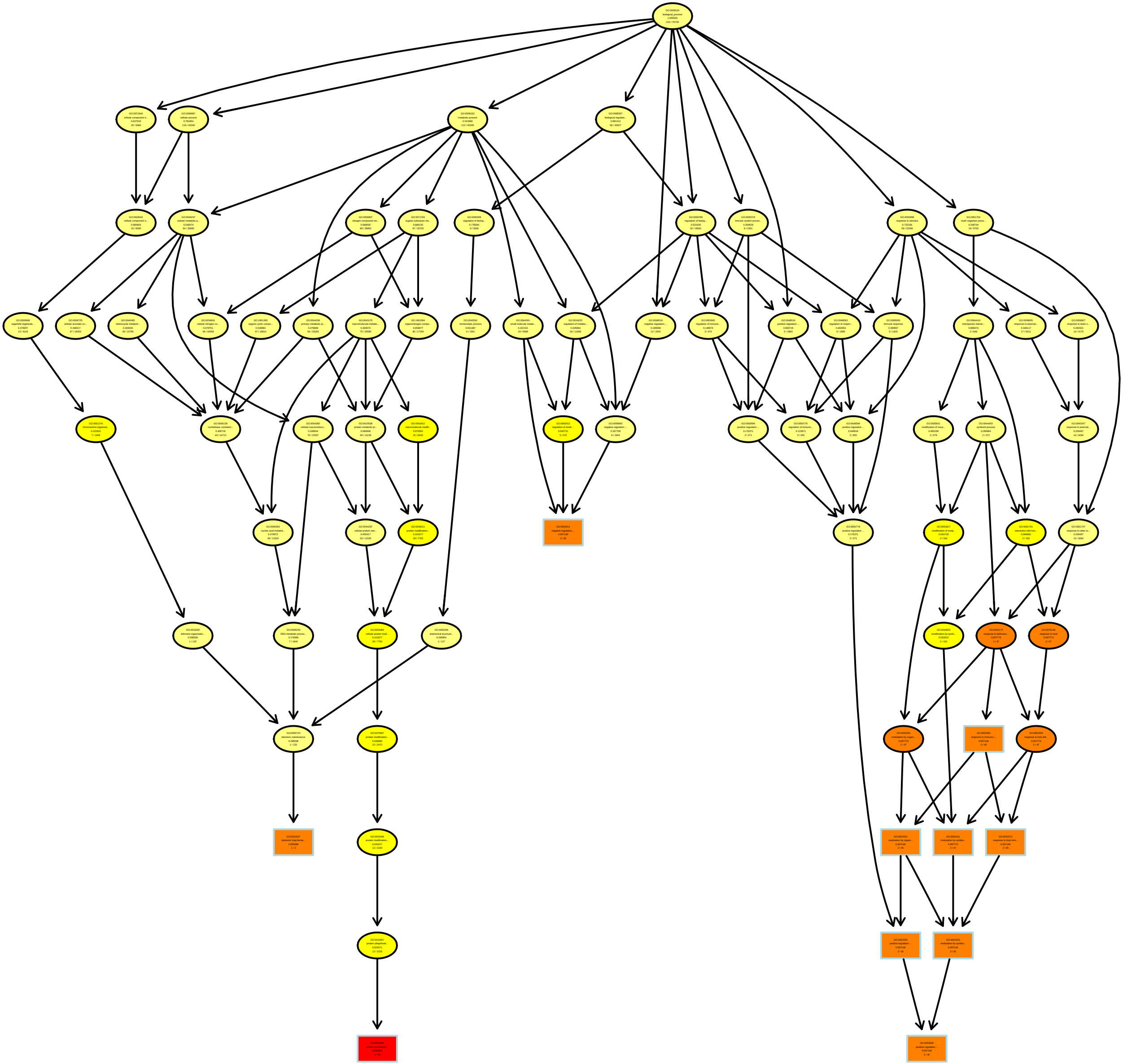

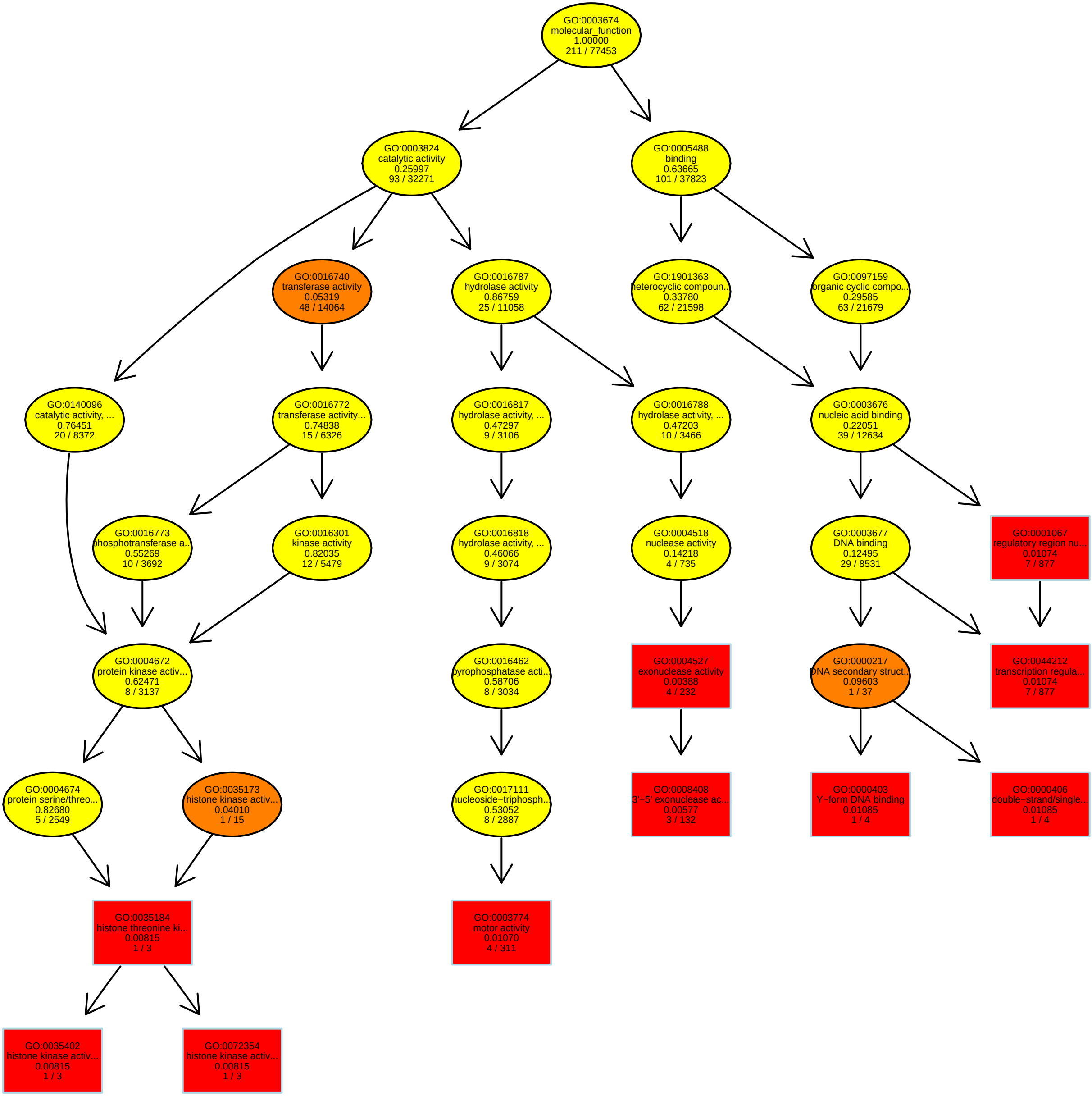
GO:0003674
molecular_function
1.00000
211 / 77453
GO:0003824
catalytic activity
0.25997
93 / 32271
GO:0005488
binding
0.63665
101 / 37823
GO:0016740
transferase activity
0.05319
48 / 14064
GO:0016787
hydrolase activity
0.86759
25 / 11058
GO:1901363
heterocyclic compoun...
0.33780
62 / 21598
GO:0097159
organic cyclic compo...
0.29585
63 / 21679
GO:0140096
catalytic activity, ...
0.76451
20 / 8372
GO:0016772
transferase activity...
0.74838
15 / 6326
GO:0016817
hydrolase activity, ...
0.47297
9 / 3106
GO:0016788
hydrolase activity, ...
0.47203
10 / 3466
GO:0003676
nucleic acid binding
0.22051
39 / 12634
GO:0016773
phosphotransferase a...
0.55269
10 / 3692
GO:0016301
kinase activity
0.82035
12 / 5479
GO:0016818
hydrolase activity, ...
0.46066
9 / 3074
GO:0004518
nuclease activity
0.14218
4 / 735
GO:0003677
DNA binding
0.12495
29 / 8531
GO:0001067
regulatory region nu...
0.01074
7 / 877
GO:0004672
protein kinase activ...
0.62471
8 / 3137
GO:0016462
pyrophosphatase acti...
0.58706
8 / 3034
GO:0004527
exonuclease activity
0.00388
4 / 232
GO:0000217
DNA secondary struct...
0.09603
1 / 37
GO:0044212
transcription regula...
0.01074
7 / 877
GO:0004674
protein serine/threo...
0.82680
5 / 2549
GO:0035173
histone kinase activ...
0.04010
1 / 15
GO:0017111
nucleoside−triphosph...
0.53052
8 / 2887
GO:0008408
3'−5' exonuclease ac...
0.00577
3 / 132
GO:0000403
Y−form DNA binding
0.01085
1 / 4
GO:0000406
double−strand/single...
0.01085
1 / 4
GO:0035184
histone threonine ki...
0.00815
1 / 3
GO:0003774
motor activity
0.01070
4 / 311
GO:0035402
histone kinase activ...
0.00815
1 / 3
GO:0072354
histone kinase activ...
0.00815
1 / 3

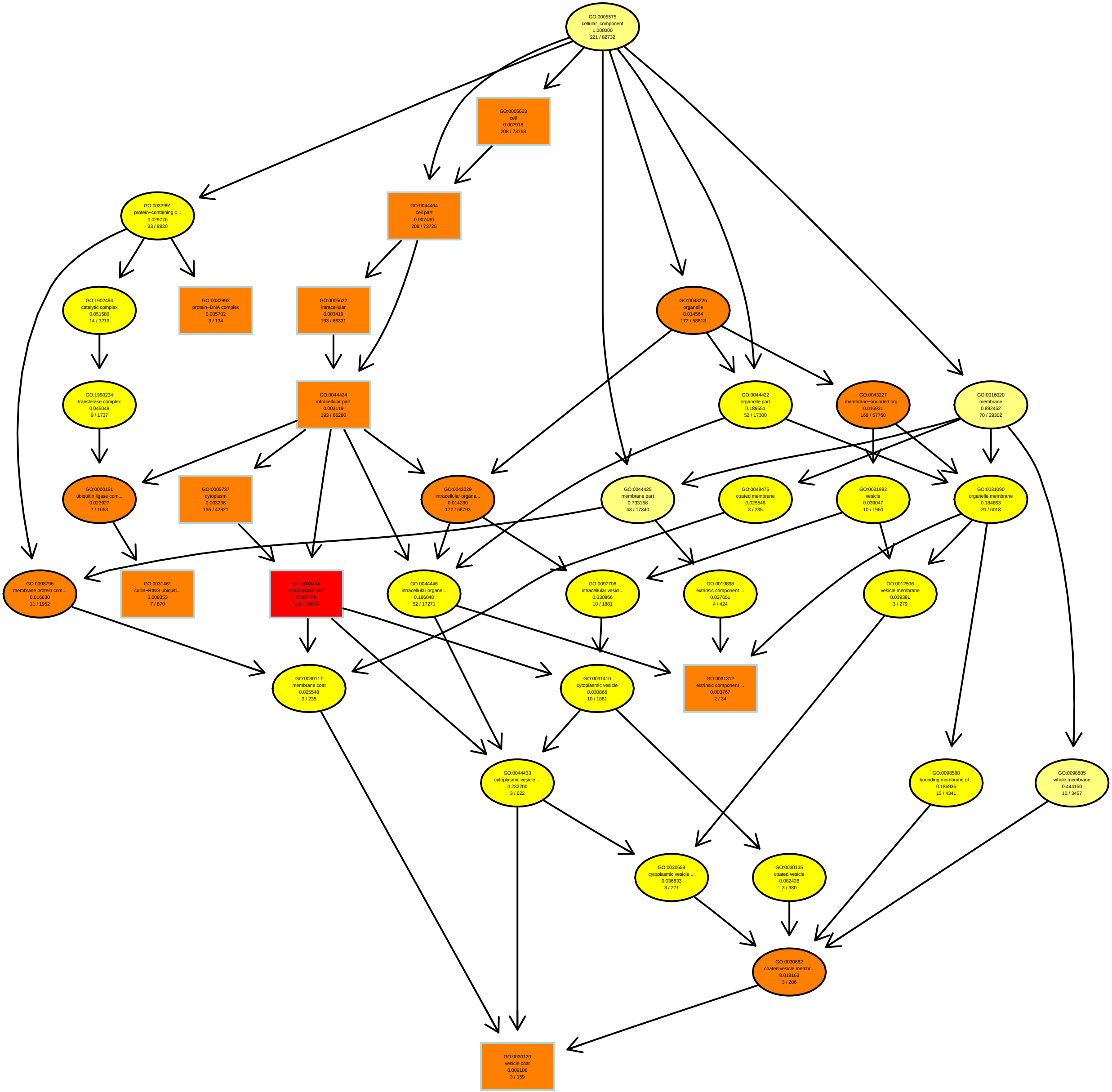

GO:0005575
cellular_component
1.000000
221 / 82732
GO:0005623
cell
0.007918
208 / 73768
GO:0032991
protein−containing c...
0.029776
33 / 8820
GO:0044464
cell part
0.007430
208 / 73725
GO:1902494
catalytic complex
0.051580
14 / 3219
GO:0032993
protein−DNA complex
0.005702
3 / 134
GO:0005622
intracellular
0.003419
193 / 66331
GO:0043226
organelle
0.014564
172 / 58813
GO:1990234
transferase complex
0.045048
9 / 1737
GO:0044424
intracellular part
0.003119
193 / 66260
GO:0044422
organelle part
0.189551
52 / 17300
GO:0043227
membrane−bounded org...
0.016921
169 / 57760
GO:0016020
membrane
0.892452
70 / 29302
GO:0000151
ubiquitin ligase com...
0.023927
7 / 1053
GO:0005737
cytoplasm
0.003236
135 / 42821
GO:0043229
intracellular organe...
0.014280
172 / 58793
GO:0044425
membrane part
0.733158
43 / 17340
GO:0048475
coated membrane
0.025548
3 / 235
GO:0031982
vesicle
0.039047
10 / 1960
GO:0031090
organelle membrane
0.184853
20 / 6018
GO:0098796
membrane protein com...
0.016630
11 / 1952
GO:0031461
cullin−RING ubiquiti...
0.009353
7 / 870
GO:0044444
cytoplasmic part
0.000788
115 / 34514
GO:0044446
intracellular organe...
0.186040
52 / 17271
GO:0097708
intracellular vesicl...
0.030866
10 / 1881
GO:0019898
extrinsic component ...
0.027651
4 / 424
GO:0012506
vesicle membrane
0.039381
3 / 279
GO:0030117
membrane coat
0.025548
3 / 235
GO:0031410
cytoplasmic vesicle
0.030866
10 / 1881
GO:0031312
extrinsic component ...
0.003767
2 / 34
GO:0044433
cytoplasmic vesicle ...
0.232200
3 / 622
GO:0098588
bounding membrane of...
0.186936
15 / 4341
GO:0098805
whole membrane
0.444150
10 / 3457
GO:0030659
cytoplasmic vesicle ...
0.036633
3 / 271
GO:0030135
coated vesicle
0.082426
3 / 380
GO:0030662
coated vesicle membr...
0.018163
3 / 206
GO:0030120
vesicle coat
0.009106
3 / 159
