# Supplementary material for: The Alternative Splicing Landscape of Brassica napus Infected with Leptosphaeria maculans
Source: Genes (Basel). 2019 Apr 11;10(4):296. doi: 10.3390/genes10040296 (PMC6523698; doi:10.3390/genes10040296)
Supplement: Supplementary file 1 [file genes-10-00296-s001.zip › Fig. S1.zip!/Fig. S1-5.pdf]

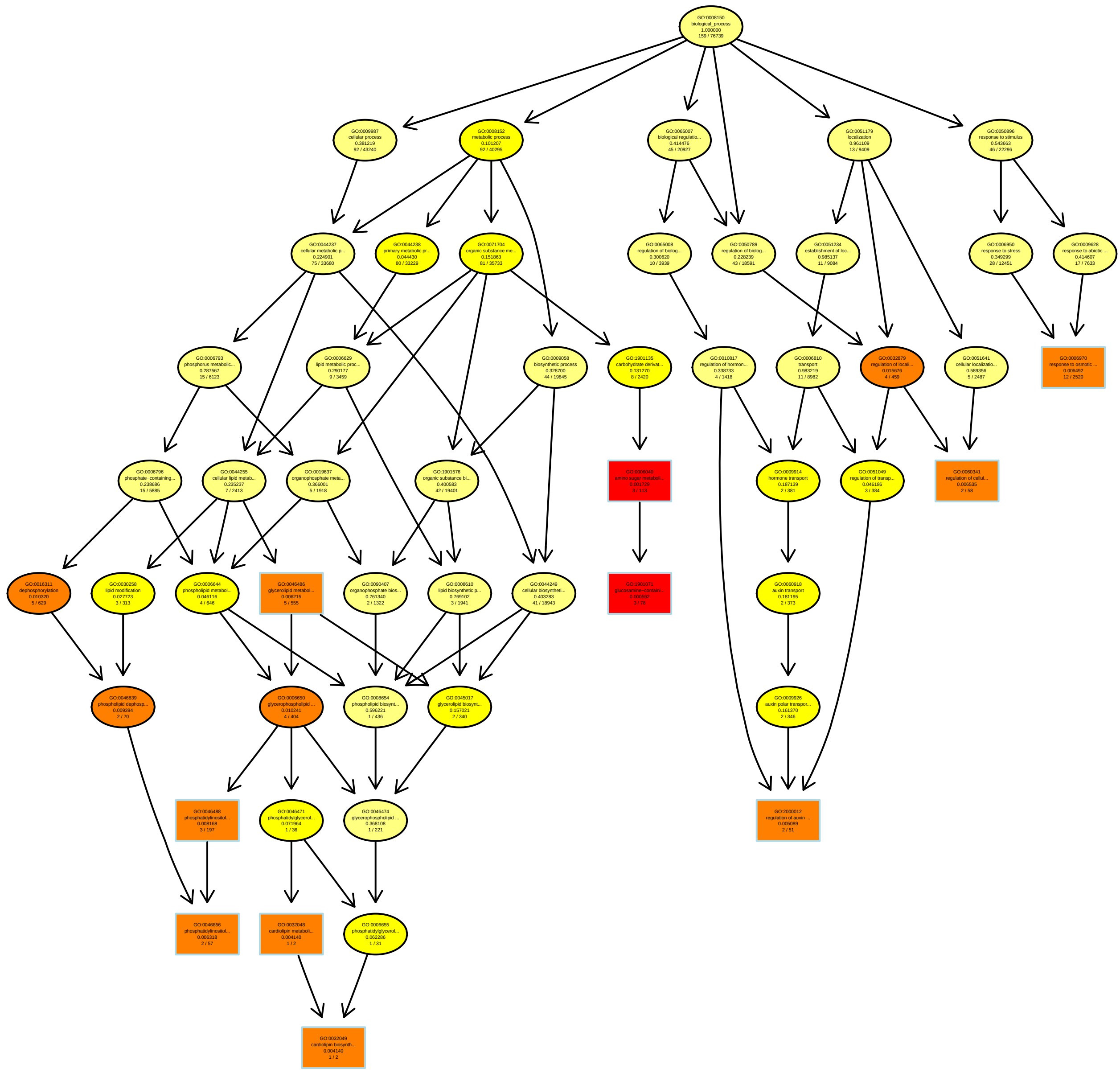

GO:0008150 biological_process 1.000000 159 / 76739
GO:0009987 cellular process 0.381219 92 / 43240
GO:0008152 metabolic process 0.101207 92 / 40295
GO:0065007 biological regulatio... 0.414476 45 / 20927
GO:0051179 localization 0.961109 13 / 9409
GO:0050896 response to stimulus 0.543663 46 / 22296
GO:0044237 cellular metabolic p... 0.224901 75 / 33680
GO:0044238 primary metabolic pr... 0.044430 80 / 33229
GO:0071704 organic substance me... 0.151863 81 / 35733
GO:0065008 regulation of biolog... 0.300620 10 / 3939
GO:0050789 regulation of biolog... 0.228239 43 / 18591
GO:0051234 establishment of loc... 0.985137 11 / 9084
GO:0006950 response to stress 0.349299 28 / 12451
GO:0009628 response to abiotic ... 0.414607 17 / 7633
GO:0006793 phosphorus metabolic... 0.287567 15 / 6123
GO:0006629 lipid metabolic proc... 0.290177 9 / 3459
GO:0009058 biosynthetic process 0.328700 44 / 19845
GO:1901135 carbohydrate derivat... 0.131270 8 / 2420
GO:0010817 regulation of hormon... 0.338733 4 / 1418
GO:0006810 transport 0.983219 11 / 8982
GO:0032879 regulation of locali... 0.015676 4 / 459
GO:0051641 cellular localizatio... 0.589356 5 / 2487
GO:0006970 response to osmotic ... 0.006492 12 / 2520
GO:0006796 phosphate−containing... 0.238686 15 / 5885
GO:0044255 cellular lipid metab... 0.235237 7 / 2413
GO:0019637 organophosphate meta... 0.366001 5 / 1918
GO:1901576 organic substance bi... 0.400583 42 / 19401
GO:0006040 amino sugar metaboli... 0.001729 3 / 113
GO:0009914 hormone transport 0.187139 2 / 381
GO:0051049 regulation of transp... 0.046186 3 / 384
GO:0060341 regulation of cellul... 0.006535 2 / 58
GO:0016311 dephosphorylation 0.010320 5 / 629
GO:0030258 lipid modification 0.027723 3 / 313
GO:0006644 phospholipid metabol... 0.046116 4 / 646
GO:0046486 glycerolipid metabol... 0.006215 5 / 555
GO:0090407 organophosphate bios... 0.761340 2 / 1322
GO:0008610 lipid biosynthetic p... 0.769102 3 / 1941
GO:0044249 cellular biosyntheti... 0.403283 41 / 18943
GO:1901071 glucosamine−containi... 0.000592 3 / 78
GO:0060918 auxin transport 0.181195 2 / 373
GO:0046839 phospholipid dephosp... 0.009394 2 / 70
GO:0006650 glycerophospholipid ... 0.010241 4 / 404
GO:0008654 phospholipid biosynt... 0.596221 1 / 436
GO:0045017 glycerolipid biosynt... 0.157021 2 / 340
GO:0009926 auxin polar transpor... 0.161370 2 / 346
GO:0046488 phosphatidylinositol... 0.008168 3 / 197
GO:0046471 phosphatidylglycerol... 0.071964 1 / 36
GO:0046474 glycerophospholipid ... 0.368108 1 / 221
GO:2000012 regulation of auxin ... 0.005089 2 / 51
GO:0046856 phosphatidylinositol... 0.006318 2 / 57
GO:0032048 cardiolipin metaboli... 0.004140 1 / 2
GO:0006655 phosphatidylglycerol... 0.062286 1 / 31
GO:0032049 cardiolipin biosynth... 0.004140 1 / 2

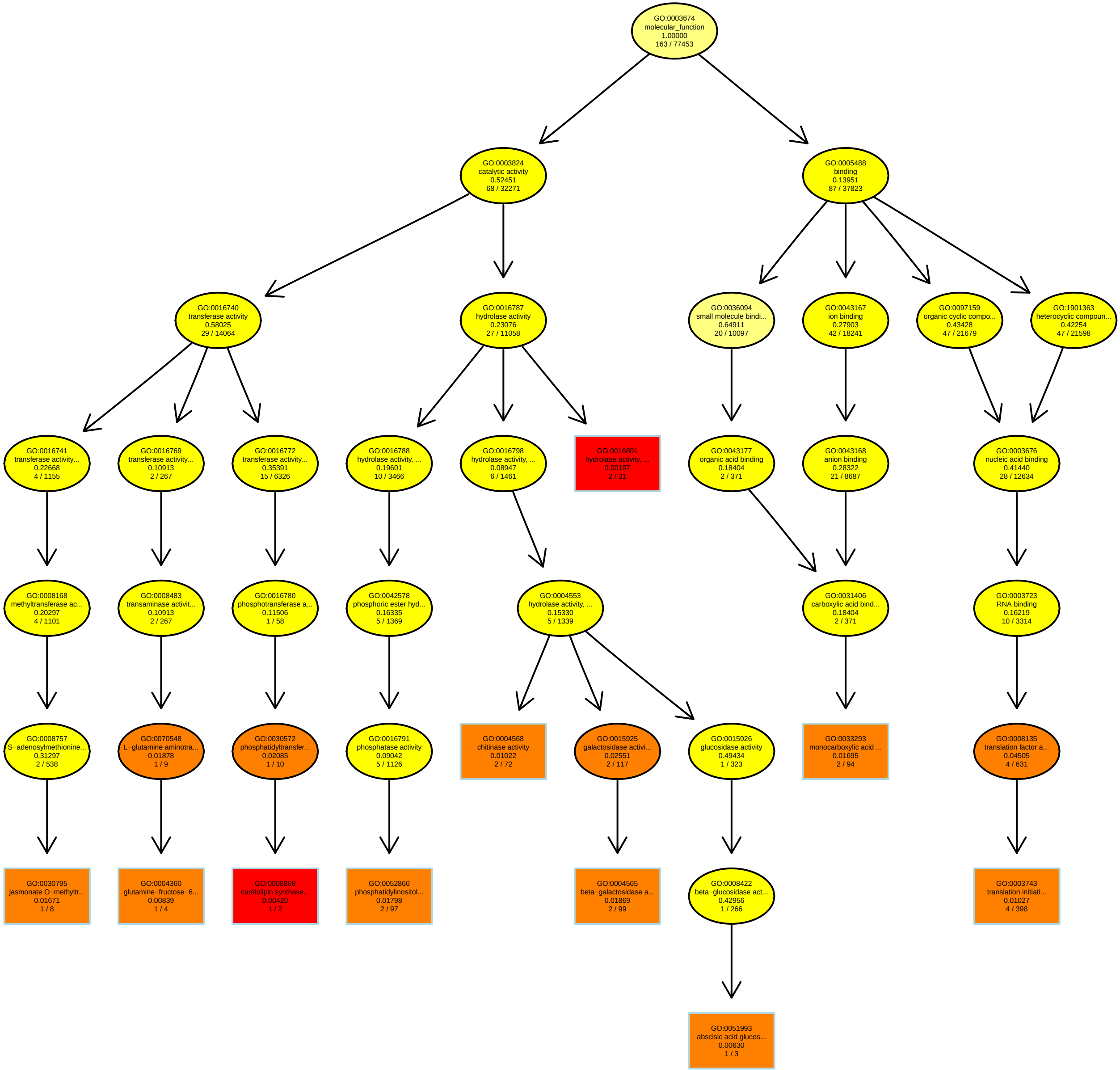
GO:0003674
molecular_function
1.00000
163 / 77453
GO:0003824
catalytic activity
0.52451
68 / 32271
GO:0005488
binding
0.13951
87 / 37823
GO:0016740
transferase activity
0.58025
29 / 14064
GO:0016787
hydrolase activity
0.23076
27 / 11058
GO:0036094
small molecule bindi...
0.64911
20 / 10097
GO:0043167
ion binding
0.27903
42 / 18241
GO:0097159
organic cyclic compo...
0.43428
47 / 21679
GO:1901363
heterocyclic compoun...
0.42254
47 / 21598
GO:0016741
transferase activity...
0.22668
4 / 1155
GO:0016769
transferase activity...
0.10913
2 / 267
GO:0016772
transferase activity...
0.35391
15 / 6326
GO:0016788
hydrolase activity, ...
0.19601
10 / 3466
GO:0016798
hydrolase activity, ...
0.08947
6 / 1461
GO:0016801
hydrolase activity, ...
0.00197
2 / 31
GO:0043177
organic acid binding
0.18404
2 / 371
GO:0043168
anion binding
0.28322
21 / 8687
GO:0003676
nucleic acid binding
0.41440
28 / 12634
GO:0008168
methyltransferase ac...
0.20297
4 / 1101
GO:0008483
transaminase activit...
0.10913
2 / 267
GO:0016780
phosphotransferase a...
0.11506
1 / 58
GO:0042578
phosphoric ester hyd...
0.16335
5 / 1369
GO:0004553
hydrolase activity, ...
0.15330
5 / 1339
GO:0031406
carboxylic acid bind...
0.18404
2 / 371
GO:0003723
RNA binding
0.16219
10 / 3314
GO:0008757
S−adenosylmethionine...
0.31297
2 / 538
GO:0070548
L−glutamine aminotra...
0.01878
1 / 9
GO:0030572
phosphatidyltransfer...
0.02085
1 / 10
GO:0016791
phosphatase activity
0.09042
5 / 1126
GO:0004568
chitinase activity
0.01022
2 / 72
GO:0015925
galactosidase activi...
0.02551
2 / 117
GO:0015926
glucosidase activity
0.49434
1 / 323
GO:0033293
monocarboxylic acid ...
0.01695
2 / 94
GO:0008135
translation factor a...
0.04505
4 / 631
GO:0030795
jasmonate O−methyltr...
0.01671
1 / 8
GO:0004360
glutamine−fructose−6...
0.00839
1 / 4
GO:0008808
cardiolipin synthase...
0.00420
1 / 2
GO:0052866
phosphatidylinositol...
0.01798
2 / 97
GO:0004565
beta−galactosidase a...
0.01869
2 / 99
GO:0008422
beta−glucosidase act...
0.42956
1 / 266
GO:0003743
translation initiati...
0.01027
4 / 398
GO:0051993
abscisic acid glucos...
0.00630
1 / 3

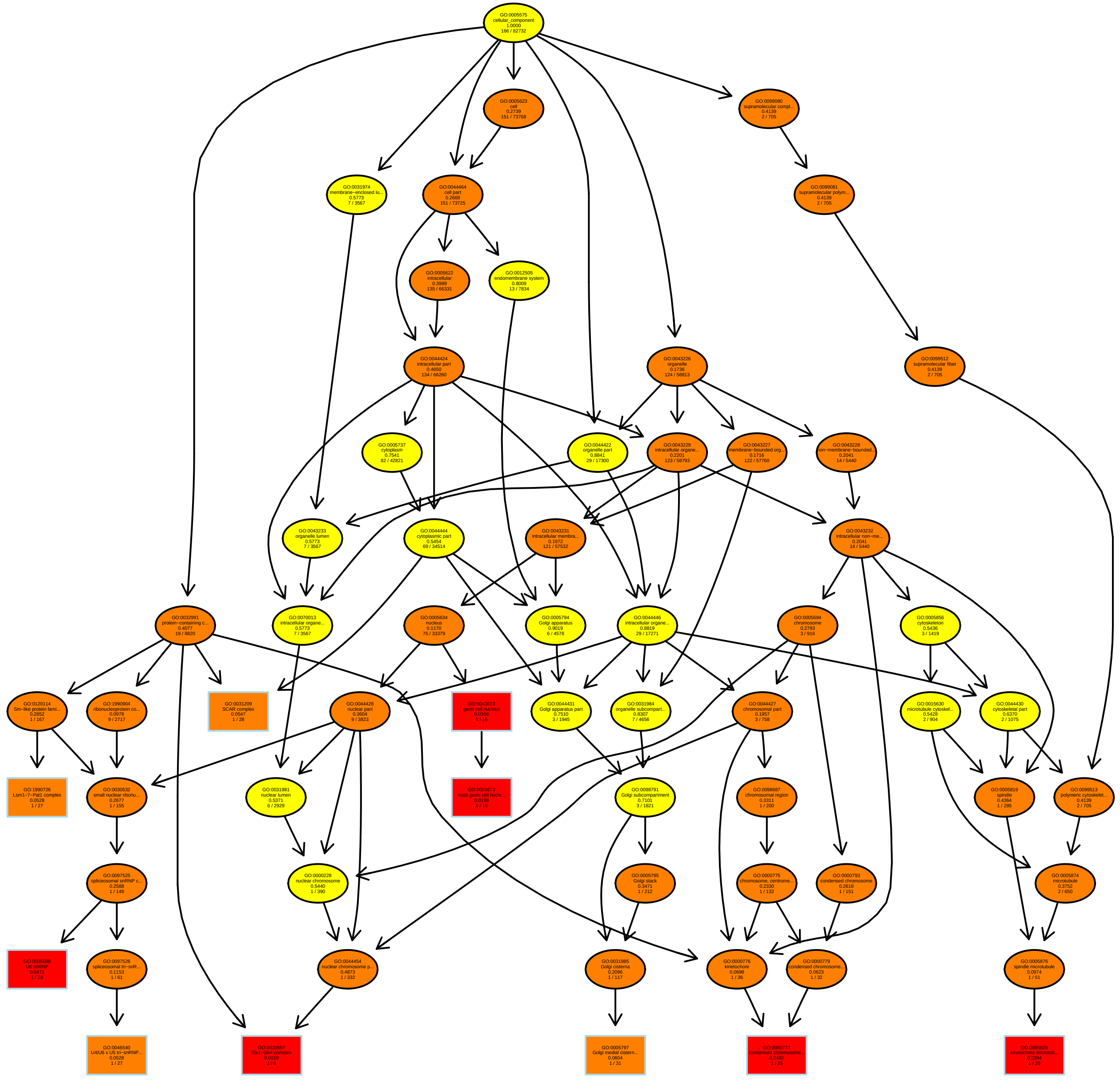

GO:0005575 cellular_component 1.0000 166 / 82732
GO:0005623 cell 0.2739 151 / 73768
GO:0099080 supramolecular compl... 0.4139 2 / 705
GO:0031974 membrane−enclosed lu... 0.5773 7 / 3567
GO:0044464 cell part 0.2669 151 / 73725
GO:0099081 supramolecular polym... 0.4139 2 / 705
GO:0005622 intracellular 0.3989 135 / 66331
GO:0012505 endomembrane system 0.8009 13 / 7834
GO:0044424 intracellular part 0.4650 134 / 66260
GO:0043226 organelle 0.1736 124 / 58813
GO:0099512 supramolecular fiber 0.4139 2 / 705
GO:0005737 cytoplasm 0.7541 82 / 42821
GO:0044422 organelle part 0.8841 29 / 17300
GO:0043229 intracellular organe... 0.2201 123 / 58793
GO:0043227 membrane−bounded org... 0.1716 122 / 57760
GO:0043228 non−membrane−bounded... 0.2041 14 / 5440
GO:0043233 organelle lumen 0.5773 7 / 3567
GO:0044444 cytoplasmic part 0.5454 69 / 34514
GO:0043231 intracellular membra... 0.1972 121 / 57532
GO:0043232 intracellular non−me... 0.2041 14 / 5440
GO:0032991 protein−containing c... 0.4077 19 / 8820
GO:0070013 intracellular organe... 0.5773 7 / 3567
GO:0005634 nucleus 0.1170 75 / 33379
GO:0005794 Golgi apparatus 0.9019 6 / 4578
GO:0044446 intracellular organe... 0.8819 29 / 17271
GO:0005694 chromosome 0.2793 3 / 916
GO:0005856 cytoskeleton 0.5436 3 / 1419
GO:0120114 Sm−like protein fami... 0.2852 1 / 167
GO:1990904 ribonucleoprotein co... 0.0978 9 / 2717
GO:0031209 SCAR complex 0.0547 1 / 28
GO:0044428 nuclear part 0.3604 9 / 3823
GO:0043073 germ cell nucleus 0.0316 1 / 16
GO:0044431 Golgi apparatus part 0.7510 3 / 1945
GO:0031984 organelle subcompart... 0.8307 7 / 4656
GO:0044427 chromosomal part 0.1957 3 / 758
GO:0015630 microtubule cytoskel... 0.5428 2 / 904
GO:0044430 cytoskeletal part 0.6370 2 / 1075
GO:1990726 Lsm1−7−Pat1 complex 0.0528 1 / 27
GO:0030532 small nuclear ribonu... 0.2677 1 / 155
GO:0031981 nuclear lumen 0.5371 6 / 2929
GO:0001673 male germ cell nucle... 0.0199 1 / 10
GO:0098791 Golgi subcompartment 0.7101 3 / 1821
GO:0098687 chromosomal region 0.3311 1 / 200
GO:0005819 spindle 0.4364 1 / 285
GO:0099513 polymeric cytoskelet... 0.4139 2 / 705
GO:0097525 spliceosomal snRNP c... 0.2588 1 / 149
GO:0000228 nuclear chromosome 0.5440 1 / 390
GO:0005795 Golgi stack 0.3471 1 / 212
GO:0000775 chromosome, centrome... 0.2330 1 / 132
GO:0000793 condensed chromosome 0.2618 1 / 151
GO:0005874 microtubule 0.3752 2 / 650
GO:0005688 U6 snRNP 0.0471 1 / 24
GO:0097526 spliceosomal tri−snR... 0.1153 1 / 61
GO:0044454 nuclear chromosome p... 0.4873 1 / 332
GO:0031985 Golgi cisterna 0.2096 1 / 117
GO:0000776 kinetochore 0.0698 1 / 36
GO:0000779 condensed chromosome... 0.0623 1 / 32
GO:0005876 spindle microtubule 0.0974 1 / 51
GO:0046540 U4/U6 x U5 tri−snRNP... 0.0528 1 / 27
GO:0033557 Slx1−Slx4 complex 0.0159 1 / 8
GO:0005797 Golgi medial cistern... 0.0604 1 / 31
GO:0000777 condensed chromosome... 0.0490 1 / 25
GO:0005828 kinetochore microtub... 0.0394 1 / 20
