# Supplementary material for: The Alternative Splicing Landscape of Brassica napus Infected with Leptosphaeria maculans
Source: Genes (Basel). 2019 Apr 11;10(4):296. doi: 10.3390/genes10040296 (PMC6523698; doi:10.3390/genes10040296)
Supplement: Supplementary file 1 [file genes-10-00296-s001.zip › Fig. S1.zip!/Fig. S1-6.pdf]

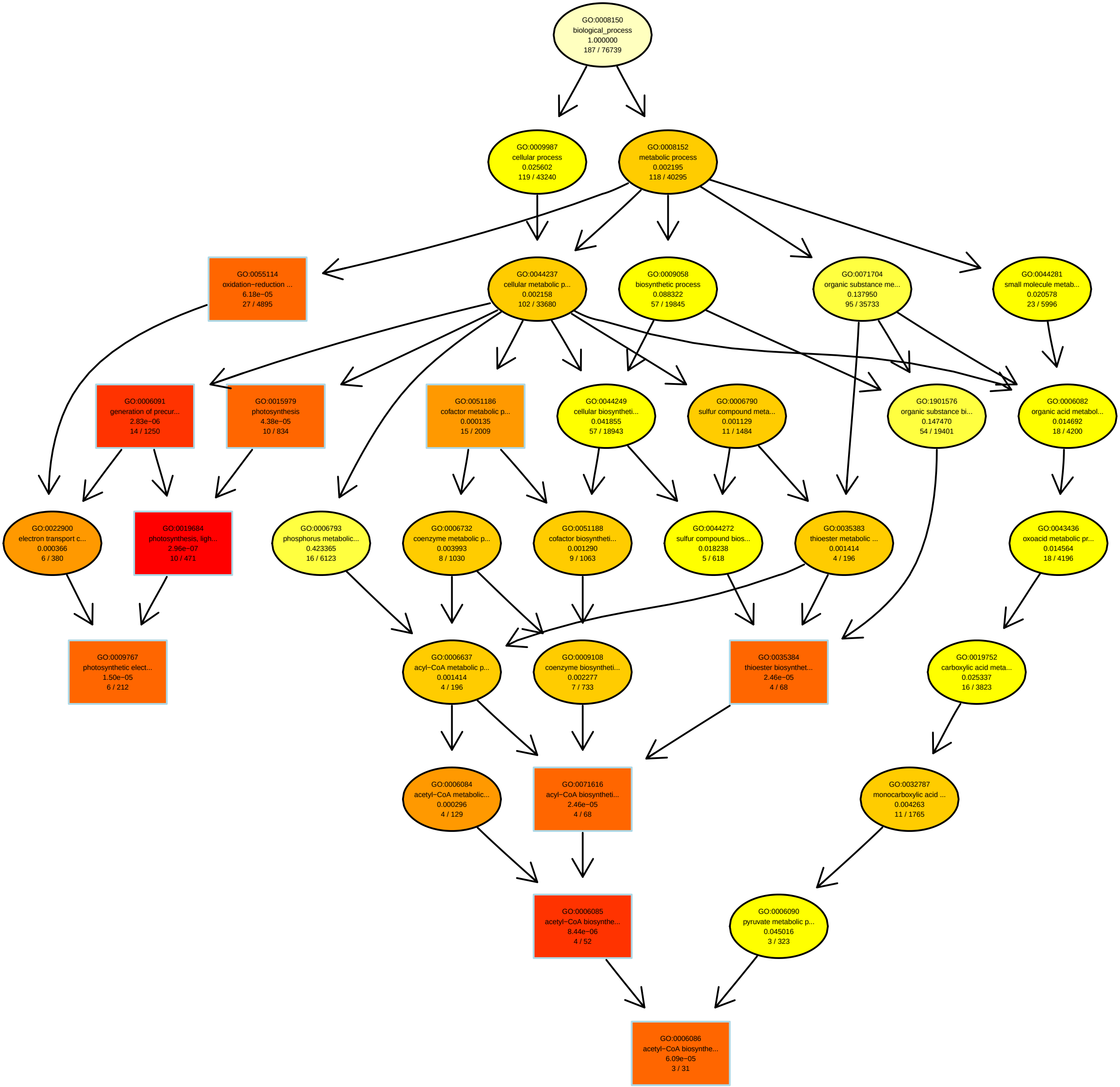
GO:0008150
biological_process
1.000000
187 / 76739
GO:0009987
cellular process
0.025602
119 / 43240
GO:0008152
metabolic process
0.002195
118 / 40295
GO:0055114
oxidation−reduction ...
6.18e−05
27 / 4895
GO:0044237
cellular metabolic p...
0.002158
102 / 33680
GO:0009058
biosynthetic process
0.088322
57 / 19845
GO:0071704
organic substance me...
0.137950
95 / 35733
GO:0044281
small molecule metab...
0.020578
23 / 5996
GO:0006091
generation of precur...
2.83e−06
14 / 1250
GO:0015979
photosynthesis
4.38e−05
10 / 834
GO:0051186
cofactor metabolic p...
0.000135
15 / 2009
GO:0044249
cellular biosyntheti...
0.041855
57 / 18943
GO:0006790
sulfur compound meta...
0.001129
11 / 1484
GO:1901576
organic substance bi...
0.147470
54 / 19401
GO:0006082
organic acid metabol...
0.014692
18 / 4200
GO:0022900
electron transport c...
0.000366
6 / 380
GO:0019684
photosynthesis, ligh...
2.96e−07
10 / 471
GO:0006793
phosphorus metabolic...
0.423365
16 / 6123
GO:0006732
coenzyme metabolic p...
0.003993
8 / 1030
GO:0051188
cofactor biosyntheti...
0.001290
9 / 1063
GO:0044272
sulfur compound bios...
0.018238
5 / 618
GO:0035383
thioester metabolic ...
0.001414
4 / 196
GO:0043436
oxoacid metabolic pr...
0.014564
18 / 4196
GO:0009767
photosynthetic elect...
1.50e−05
6 / 212
GO:0006637
acyl−CoA metabolic p...
0.001414
4 / 196
GO:0009108
coenzyme biosyntheti...
0.002277
7 / 733
GO:0035384
thioester biosynthet...
2.46e−05
4 / 68
GO:0019752
carboxylic acid meta...
0.025337
16 / 3823
GO:0006084
acetyl−CoA metabolic...
0.000296
4 / 129
GO:0071616
acyl−CoA biosyntheti...
2.46e−05
4 / 68
GO:0032787
monocarboxylic acid ...
0.004263
11 / 1765
GO:0006085
acetyl−CoA biosynthe...
8.44e−06
4 / 52
GO:0006090
pyruvate metabolic p...
0.045016
3 / 323
GO:0006086
acetyl−CoA biosynthe...
6.09e−05
3 / 31

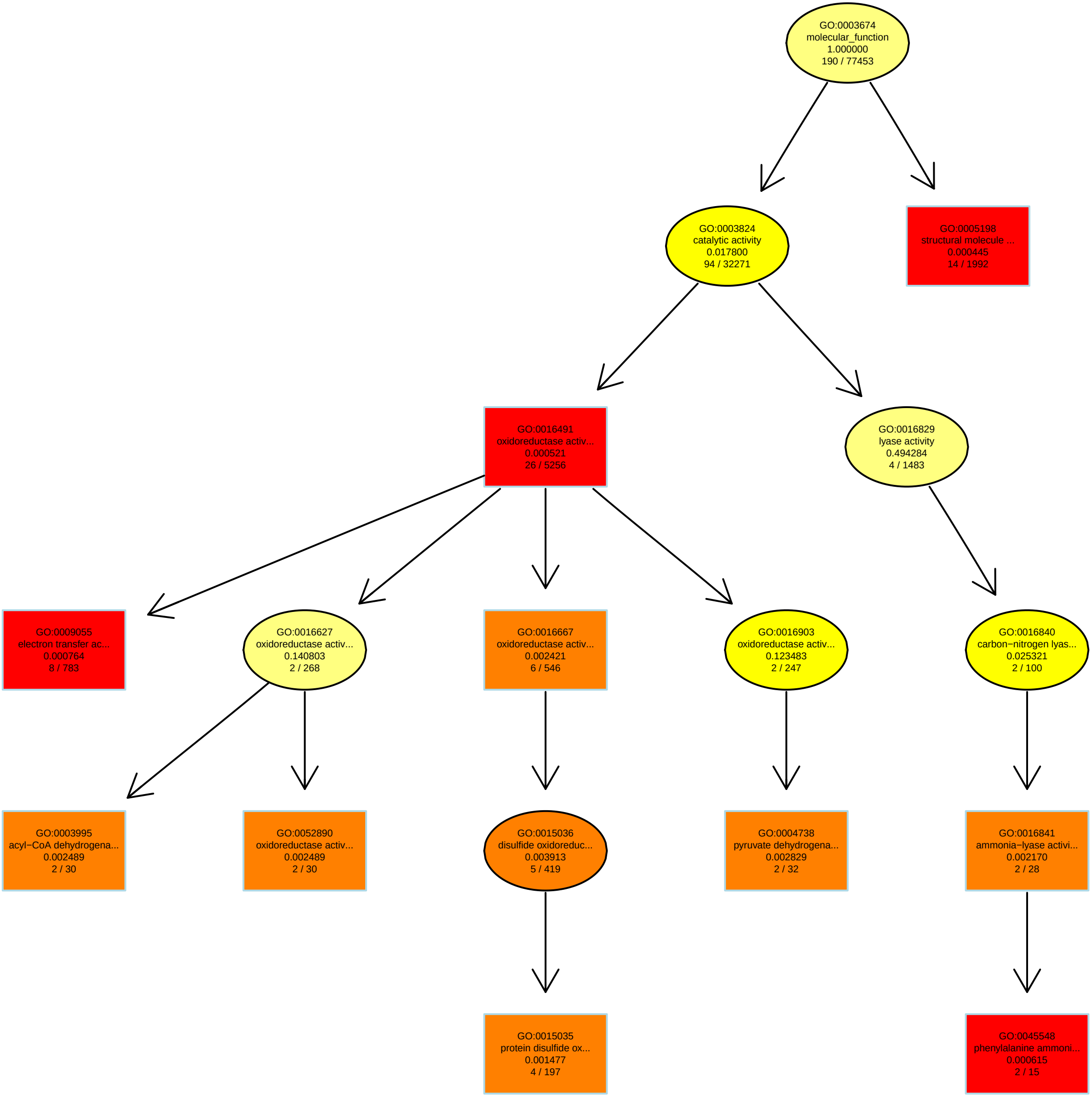
GO:0003674
molecular_function
1.000000
190 / 77453
GO:0003824
catalytic activity
0.017800
94 / 32271
GO:0005198
structural molecule ...
0.000445
14 / 1992
GO:0016491
oxidoreductase activ...
0.000521
26 / 5256
GO:0016829
lyase activity
0.494284
4 / 1483
GO:0009055
electron transfer ac...
0.000764
8 / 783
GO:0016627
oxidoreductase activ...
0.140803
2 / 268
GO:0016667
oxidoreductase activ...
0.002421
6 / 546
GO:0016903
oxidoreductase activ...
0.123483
2 / 247
GO:0016840
carbon−nitrogen lyas...
0.025321
2 / 100
GO:0003995
acyl−CoA dehydrogena...
0.002489
2 / 30
GO:0052890
oxidoreductase activ...
0.002489
2 / 30
GO:0015036
disulfide oxidoreduc...
0.003913
5 / 419
GO:0004738
pyruvate dehydrogena...
0.002829
2 / 32
GO:0016841
ammonia−lyase activi...
0.002170
2 / 28
GO:0015035
protein disulfide ox...
0.001477
4 / 197
GO:0045548
phenylalanine ammoni...
0.000615
2 / 15

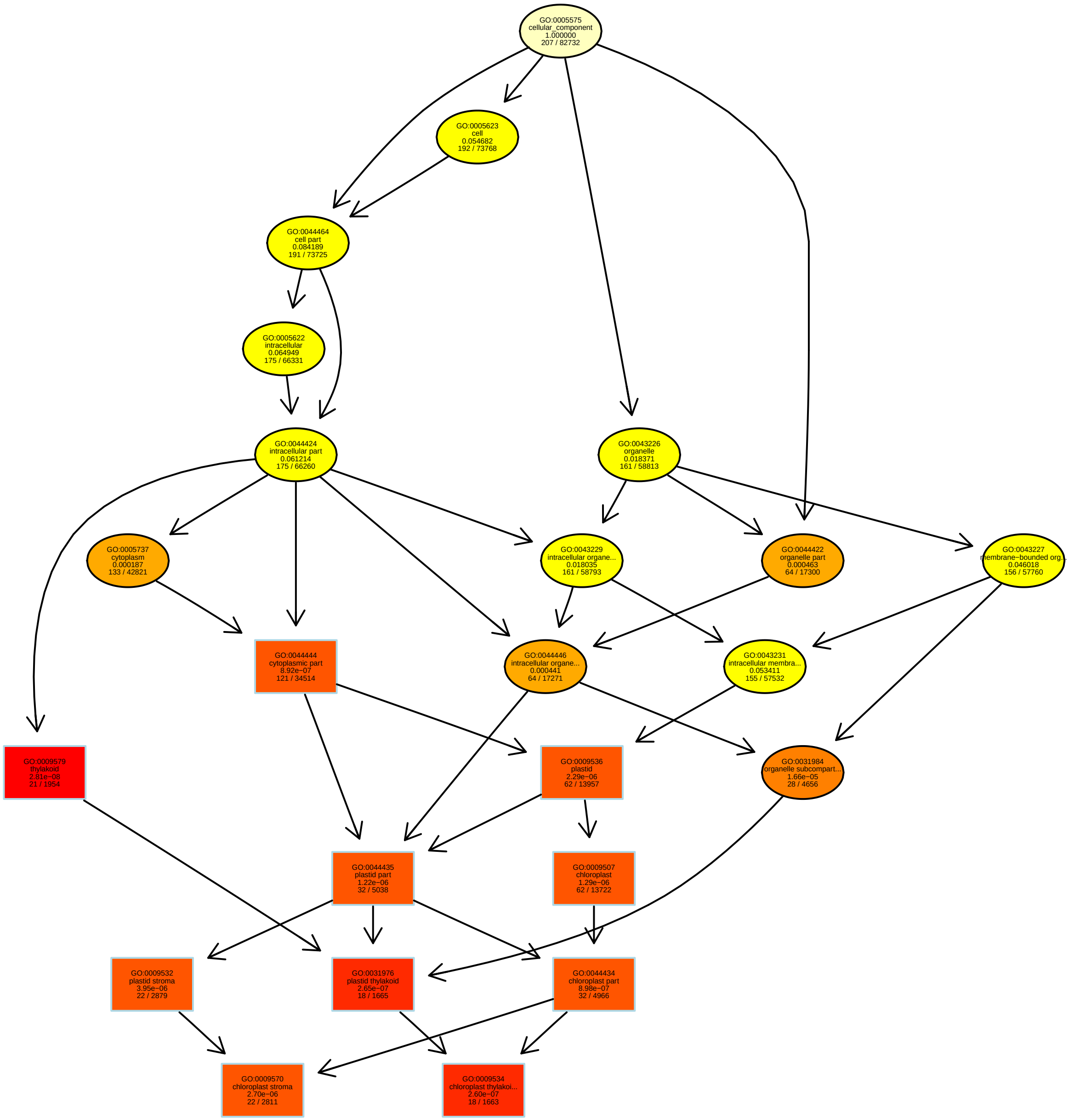
GO:0005575
cellular_component
1.000000
207 / 82732
GO:0005623
cell
0.054682
192 / 73768
GO:0044464
cell part
0.084189
191 / 73725
GO:0005622
intracellular
0.064949
175 / 66331
GO:0044424
intracellular part
0.061214
175 / 66260
GO:0043226
organelle
0.018371
161 / 58813
GO:0005737
cytoplasm
0.000187
133 / 42821
GO:0043229
intracellular organe...
0.018035
161 / 58793
GO:0044422
organelle part
0.000463
64 / 17300
GO:0043227
membrane−bounded org...
0.046018
156 / 57760
GO:0044444
cytoplasmic part
8.92e−07
121 / 34514
GO:0044446
intracellular organe...
0.000441
64 / 17271
GO:0043231
intracellular membra...
0.053411
155 / 57532
GO:0009579
thylakoid
2.81e−08
21 / 1954
GO:0009536
plastid
2.29e−06
62 / 13957
GO:0031984
organelle subcompart...
1.66e−05
28 / 4656
GO:0044435
plastid part
1.22e−06
32 / 5038
GO:0009507
chloroplast
1.29e−06
62 / 13722
GO:0009532
plastid stroma
3.95e−06
22 / 2879
GO:0031976
plastid thylakoid
2.65e−07
18 / 1665
GO:0044434
chloroplast part
8.98e−07
32 / 4966
GO:0009570
chloroplast stroma
2.70e−06
22 / 2811
GO:0009534
chloroplast thylakoi...
2.60e−07
18 / 1663
