# Supplementary material for: The Alternative Splicing Landscape of Brassica napus Infected with Leptosphaeria maculans
Source: Genes (Basel). 2019 Apr 11;10(4):296. doi: 10.3390/genes10040296 (PMC6523698; doi:10.3390/genes10040296)
Supplement: Supplementary file 1 [file genes-10-00296-s001.zip › Fig. S1.zip!/Fig. S1-7.pdf]

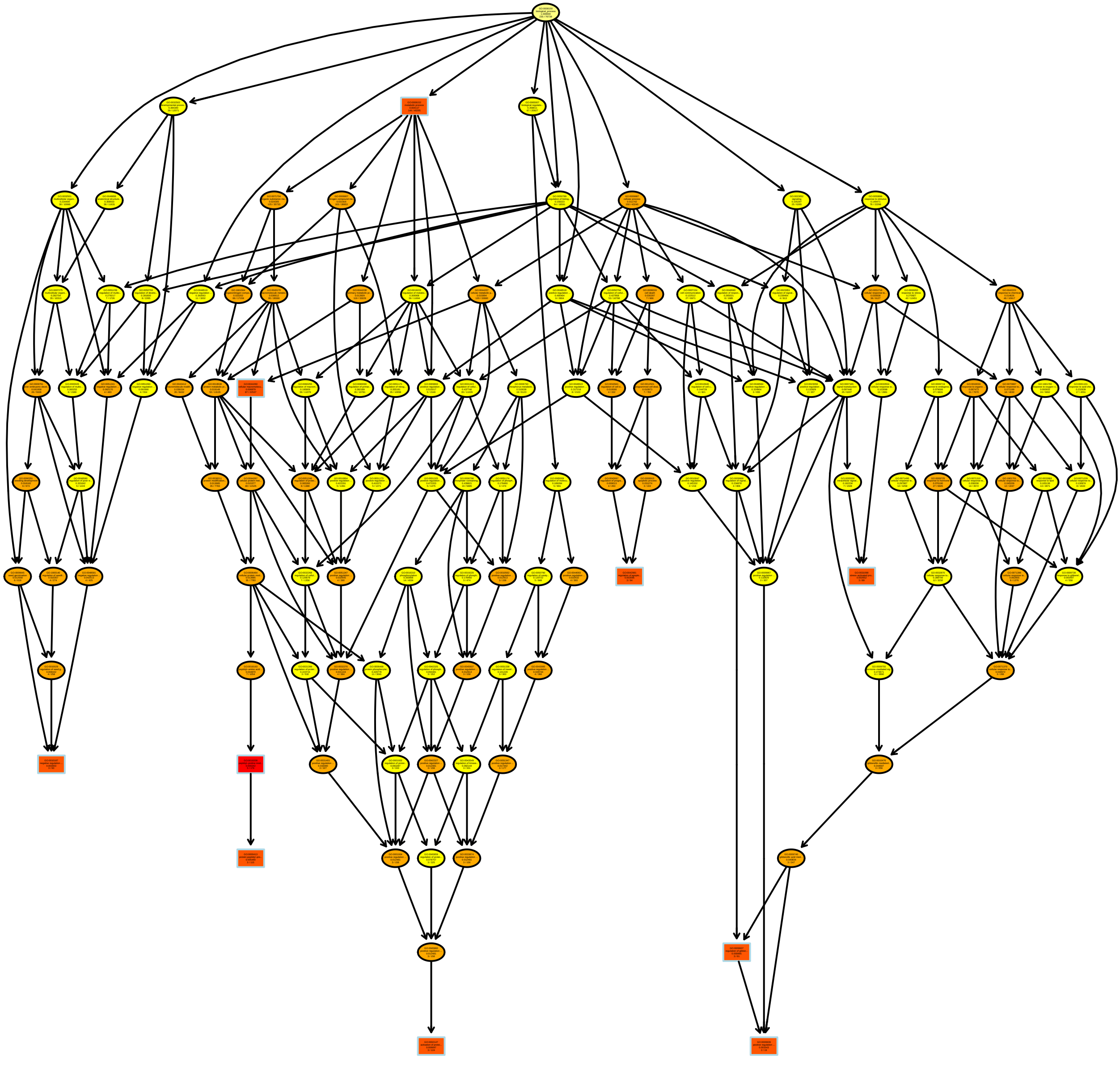

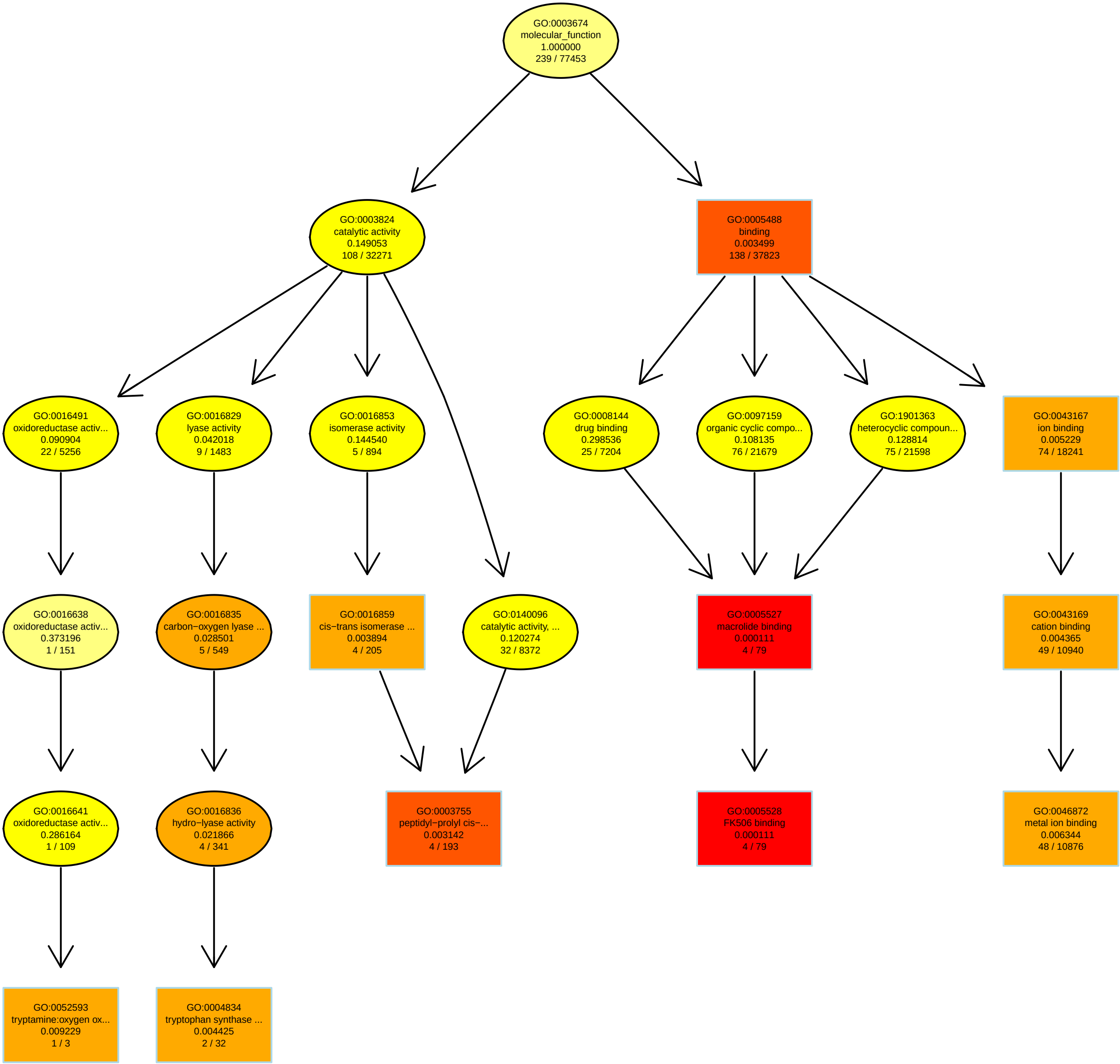
GO:0003674
molecular_function
1.000000
239 / 77453
GO:0003824
catalytic activity
0.149053
108 / 32271
GO:0005488
binding
0.003499
138 / 37823
GO:0016491
oxidoreductase activ...
0.090904
22 / 5256
GO:0016829
lyase activity
0.042018
9 / 1483
GO:0016853
isomerase activity
0.144540
5 / 894
GO:0008144
drug binding
0.298536
25 / 7204
GO:0097159
organic cyclic compo...
0.108135
76 / 21679
GO:1901363
heterocyclic compoun...
0.128814
75 / 21598
GO:0043167
ion binding
0.005229
74 / 18241
GO:0016638
oxidoreductase activ...
0.373196
1 / 151
GO:0016835
carbon−oxygen lyase ...
0.028501
5 / 549
GO:0016859
cis−trans isomerase ...
0.003894
4 / 205
GO:0140096
catalytic activity, ...
0.120274
32 / 8372
GO:0005527
macrolide binding
0.000111
4 / 79
GO:0043169
cation binding
0.004365
49 / 10940
GO:0016641
oxidoreductase activ...
0.286164
1 / 109
GO:0016836
hydro−lyase activity
0.021866
4 / 341
GO:0003755
peptidyl−prolyl cis−...
0.003142
4 / 193
GO:0005528
FK506 binding
0.000111
4 / 79
GO:0046872
metal ion binding
0.006344
48 / 10876
GO:0052593
tryptamine:oxygen ox...
0.009229
1 / 3
GO:0004834
tryptophan synthase ...
0.004425
2 / 32

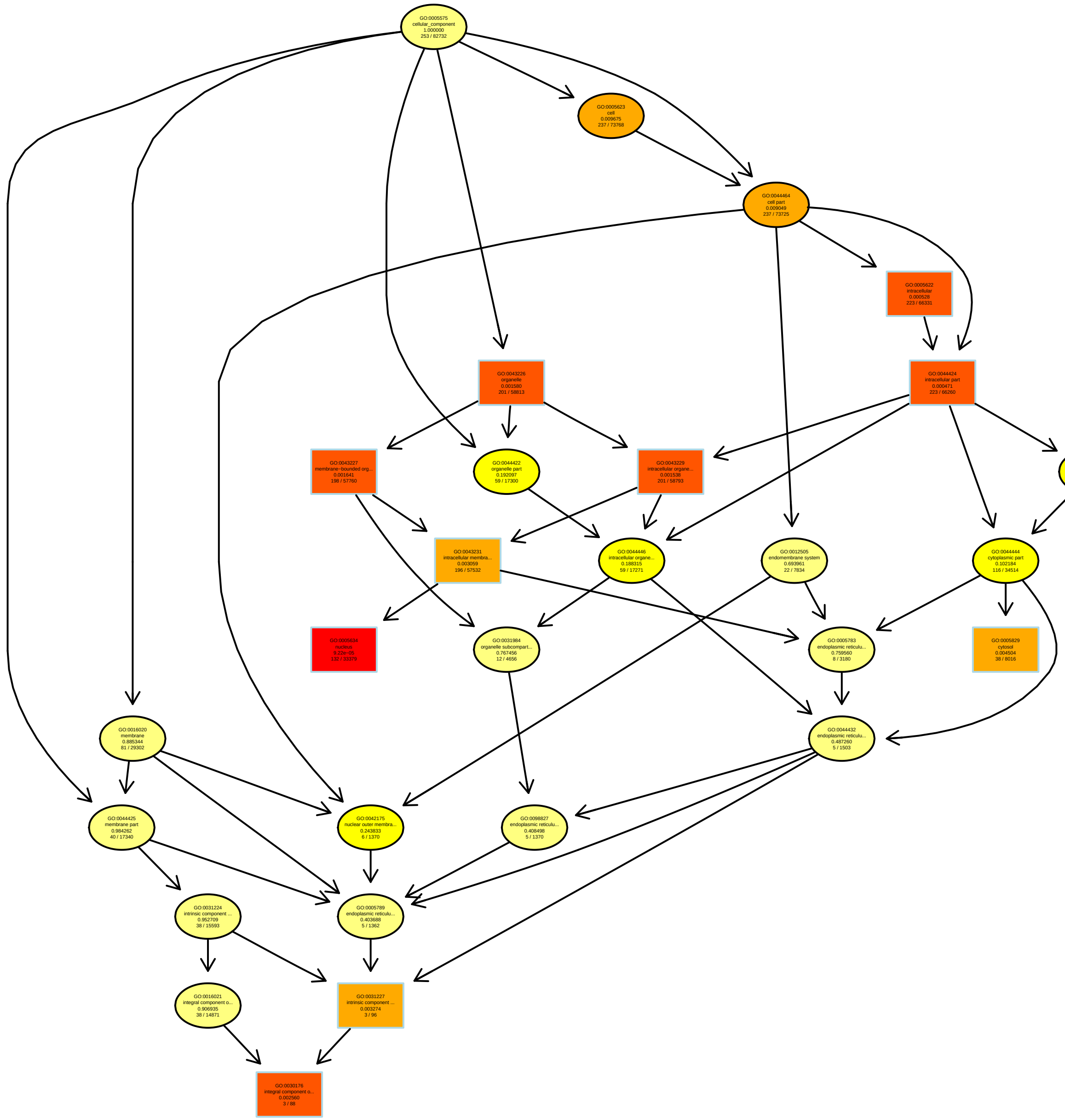
GO:0005575 cellular_component 1.000000 253 / 82732
GO:0005623 cell 0.009675 237 / 73768
GO:0044464 cell part 0.009049 237 / 73725
GO:0005622 intracellular 0.000528 223 / 66331
GO:0043226 organelle 0.001580 201 / 58813
GO:0044424 intracellular part 0.000471 223 / 66260
GO:0043227 membrane−bounded org... 0.001641 198 / 57760
GO:0044422 organelle part 0.192097 59 / 17300
GO:0043229 intracellular organe... 0.001538 201 / 58793
GO:0005737 cytoplasm 0.170717 139 / 42821
GO:0043231 intracellular membra... 0.003059 196 / 57532
GO:0044446 intracellular organe... 0.188315 59 / 17271
GO:0012505 endomembrane system 0.693961 22 / 7834
GO:0044444 cytoplasmic part 0.102184 116 / 34514
GO:0005634 nucleus 9.22e−05 132 / 33379
GO:0031984 organelle subcompart... 0.767456 12 / 4656
GO:0005783 endoplasmic reticulu... 0.759560 8 / 3180
GO:0005829 cytosol 0.004504 38 / 8016
GO:0016020 membrane 0.885344 81 / 29302
GO:0044432 endoplasmic reticulu... 0.487260 5 / 1503
GO:0044425 membrane part 0.984262 40 / 17340
GO:0042175 nuclear outer membra... 0.243833 6 / 1370
GO:0098827 endoplasmic reticulu... 0.408498 5 / 1370
GO:0031224 intrinsic component ... 0.952709 38 / 15593
GO:0005789 endoplasmic reticulu... 0.403688 5 / 1362
GO:0016021 integral component o... 0.906935 38 / 14871
GO:0031227 intrinsic component ... 0.003274 3 / 96
GO:0030176 integral component o... 0.002560 3 / 88
