# Supplementary material for: The Alternative Splicing Landscape of Brassica napus Infected with Leptosphaeria maculans
Source: Genes (Basel). 2019 Apr 11;10(4):296. doi: 10.3390/genes10040296 (PMC6523698; doi:10.3390/genes10040296)
Supplement: Supplementary file 1 [file genes-10-00296-s001.zip › Fig. S1.zip!/Fig. S1-8.pdf]

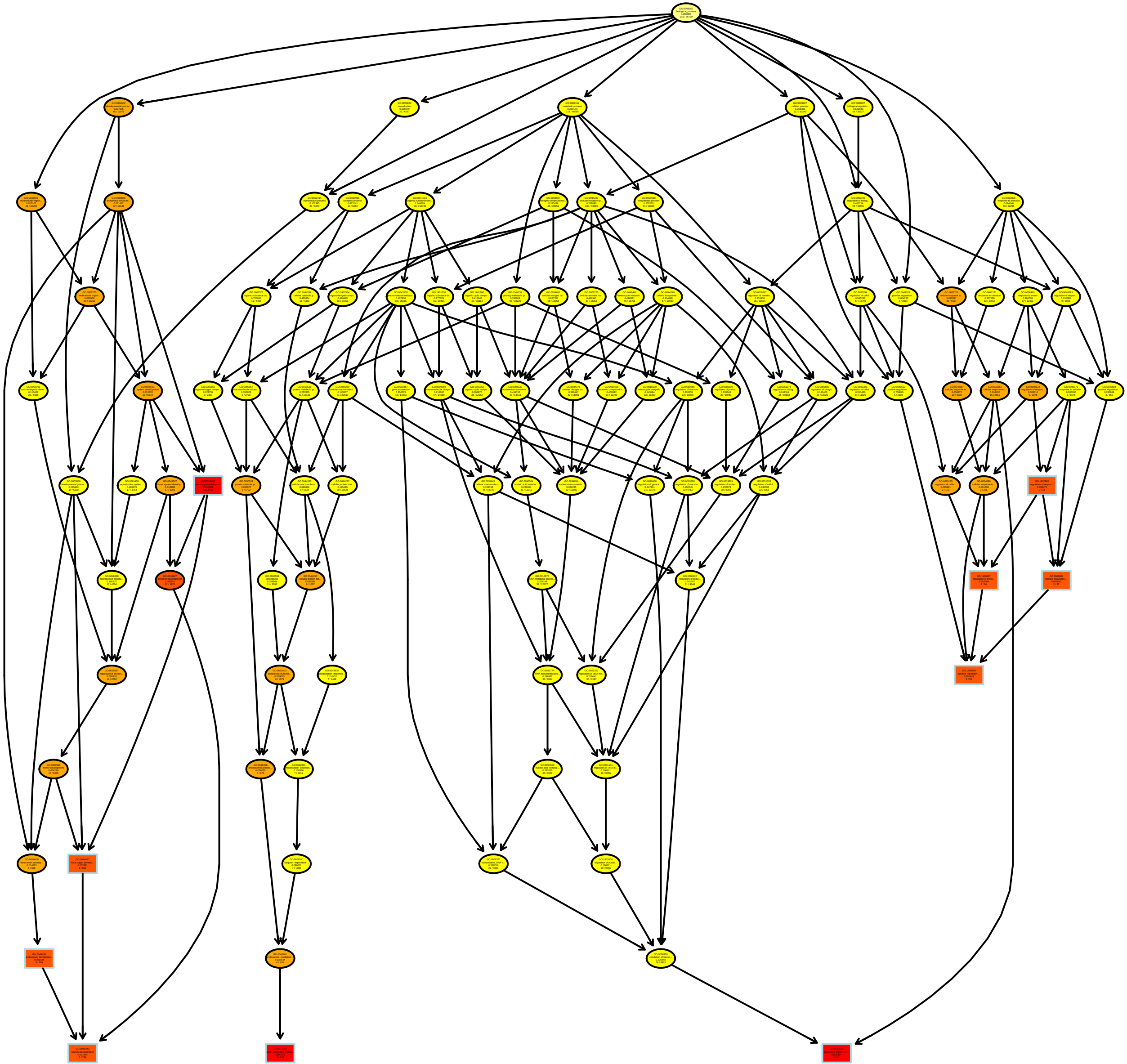

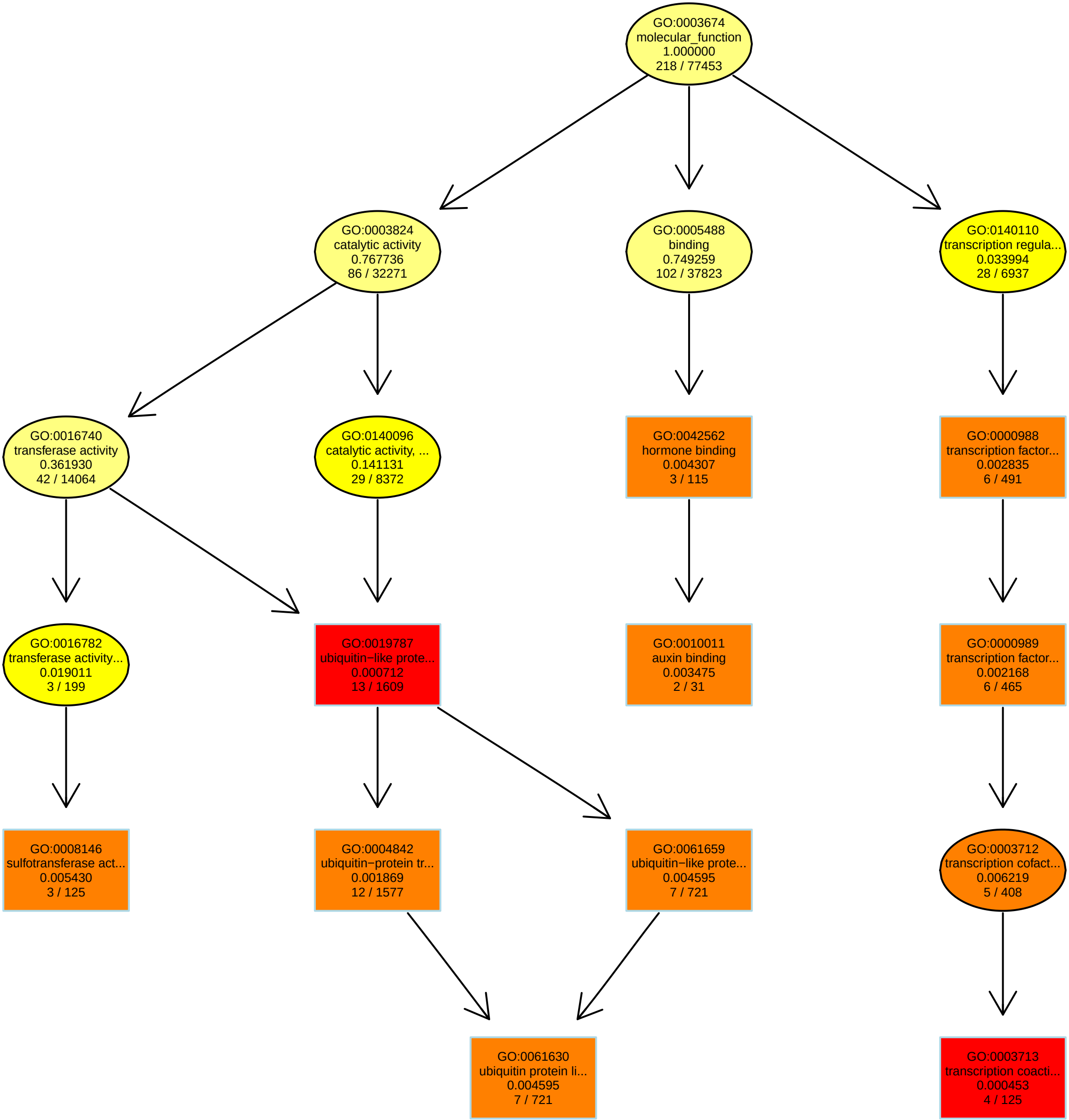
GO:0003674
molecular_function
1.000000
218 / 77453
GO:0003824
catalytic activity
0.767736
86 / 32271
GO:0005488
binding
0.749259
102 / 37823
GO:0140110
transcription regula...
0.033994
28 / 6937
GO:0016740
transferase activity
0.361930
42 / 14064
GO:0140096
catalytic activity, ...
0.141131
29 / 8372
GO:0042562
hormone binding
0.004307
3 / 115
GO:0000988
transcription factor...
0.002835
6 / 491
GO:0016782
transferase activity...
0.019011
3 / 199
GO:0019787
ubiquitin−like prote...
0.000712
13 / 1609
GO:0010011
auxin binding
0.003475
2 / 31
GO:0000989
transcription factor...
0.002168
6 / 465
GO:0008146
sulfotransferase act...
0.005430
3 / 125
GO:0004842
ubiquitin−protein tr...
0.001869
12 / 1577
GO:0061659
ubiquitin−like prote...
0.004595
7 / 721
GO:0003712
transcription cofact...
0.006219
5 / 408
GO:0061630
ubiquitin protein li...
0.004595
7 / 721
GO:0003713
transcription coacti...
0.000453
4 / 125

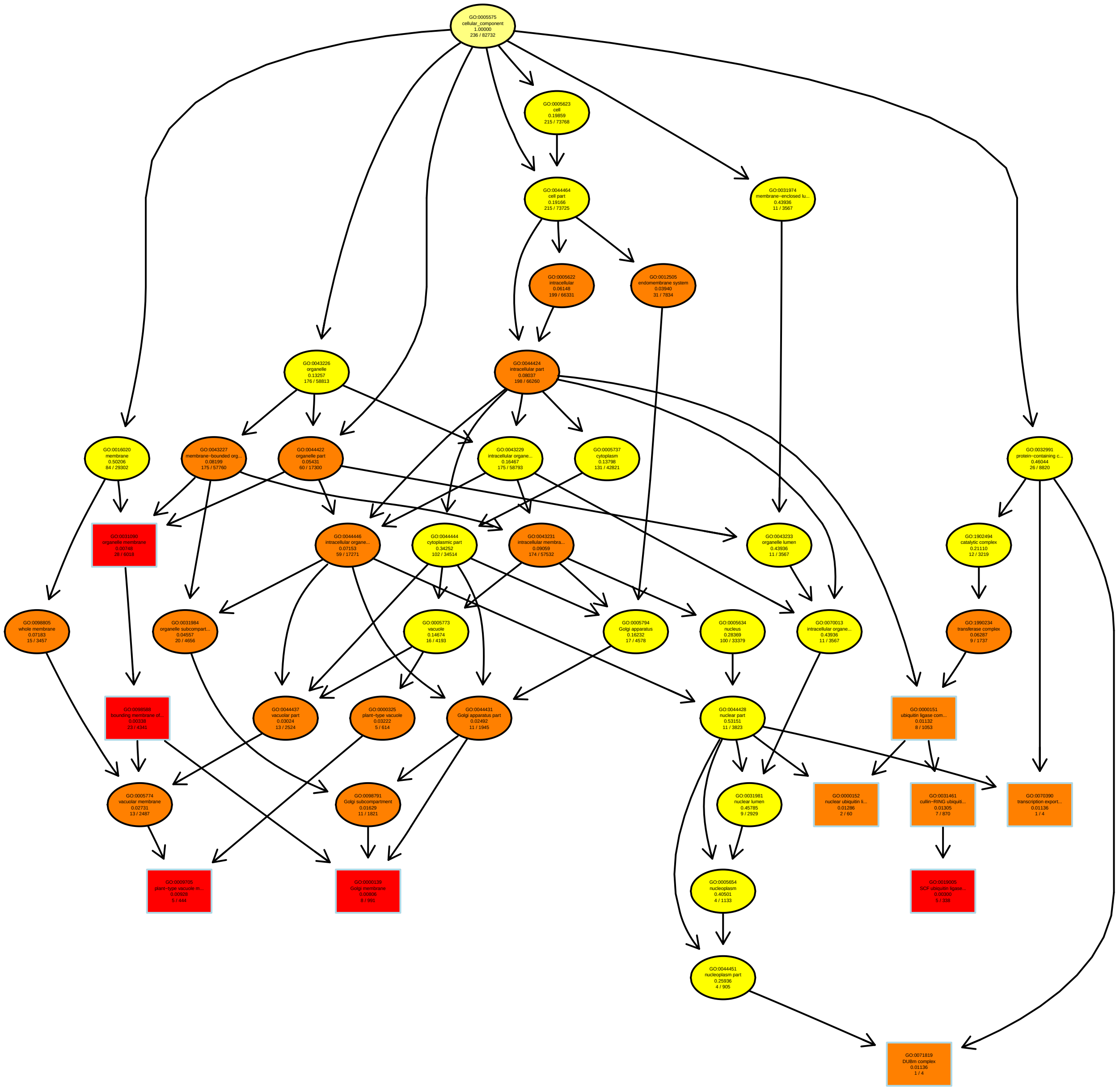

GO:0005575 cellular_component 1.00000 236 / 82732
GO:0005623 cell 0.19859 215 / 73768
GO:0044464 cell part 0.19166 215 / 73725
GO:0031974 membrane−enclosed lu... 0.43936 11 / 3567
GO:0005622 intracellular 0.06148 199 / 66331
GO:0012505 endomembrane system 0.03940 31 / 7834
GO:0043226 organelle 0.13257 176 / 58813
GO:0044424 intracellular part 0.08037 198 / 66260
GO:0016020 membrane 0.50206 84 / 29302
GO:0043227 membrane−bounded org... 0.08199 175 / 57760
GO:0044422 organelle part 0.05431 60 / 17300
GO:0043229 intracellular organe... 0.16467 175 / 58793
GO:0005737 cytoplasm 0.13798 131 / 42821
GO:0032991 protein−containing c... 0.46044 26 / 8820
GO:0031090 organelle membrane 0.00748 28 / 6018
GO:0044446 intracellular organe... 0.07153 59 / 17271
GO:0044444 cytoplasmic part 0.34252 102 / 34514
GO:0043231 intracellular membra... 0.09059 174 / 57532
GO:0043233 organelle lumen 0.43936 11 / 3567
GO:1902494 catalytic complex 0.21110 12 / 3219
GO:0098805 whole membrane 0.07183 15 / 3457
GO:0031984 organelle subcompart... 0.04557 20 / 4656
GO:0005773 vacuole 0.14674 16 / 4193
GO:0005794 Golgi apparatus 0.16232 17 / 4578
GO:0005634 nucleus 0.28369 100 / 33379
GO:0070013 intracellular organe... 0.43936 11 / 3567
GO:1990234 transferase complex 0.06287 9 / 1737
GO:0098588 bounding membrane of... 0.00338 23 / 4341
GO:0044437 vacuolar part 0.03024 13 / 2524
GO:0000325 plant−type vacuole 0.03222 5 / 614
GO:0044431 Golgi apparatus part 0.02492 11 / 1945
GO:0044428 nuclear part 0.53151 11 / 3823
GO:0000151 ubiquitin ligase com... 0.01132 8 / 1053
GO:0005774 vacuolar membrane 0.02731 13 / 2487
GO:0098791 Golgi subcompartment 0.01629 11 / 1821
GO:0031981 nuclear lumen 0.45785 9 / 2929
GO:0000152 nuclear ubiquitin li... 0.01286 2 / 60
GO:0031461 cullin−RING ubiquiti... 0.01305 7 / 870
GO:0070390 transcription export... 0.01136 1 / 4
GO:0009705 plant−type vacuole m... 0.00928 5 / 444
GO:0000139 Golgi membrane 0.00806 8 / 991
GO:0005654 nucleoplasm 0.40501 4 / 1133
GO:0019005 SCF ubiquitin ligase... 0.00300 5 / 338
GO:0044451 nucleoplasm part 0.25936 4 / 905
GO:0071819 DUBm complex 0.01136 1 / 4
